# TET exhibits enzymatic-independent and-dependent functions during *Drosophila* flight muscle development and aging

**DOI:** 10.1186/s13395-025-00399-x

**Published:** 2025-10-27

**Authors:** Vincent Gerdy, Emilie Plantié, Romane Bertrand, Yoan Renaud, Guillaume Junion, Laurence Vandel, Lucas Waltzer

**Affiliations:** Université Clermont Auvergne, CNRS, INSERM, iGReD, 63000 Clermont-Ferrand, France

**Keywords:** Ten-eleven translocation, *Drosophila*, Muscles, Flight, Mobility, Aging, Catalytic activity

## Abstract

**Background:**

Enzymes of the Ten-Eleven Translocation family are responsible for 5-methylcytosine (5mC) oxidation and play a key role in regulating DNA demethylation during various developmental processes, including myogenesis. However, they also exhibit 5mC-independent functions whose importance for muscle development remains unexplored. As the *Drosophila* genome lacks the enzymes required for 5mC deposition but contains a single *Tet* gene essential for viability, we analyzed its role in flight muscle development.

**Methods:**

Using a combination of genetics, imaging techniques, transcriptomic analysis and functional assays, we assessed the impact of *Tet* loss of function (using either *Tet null* or *Tet catalytic inactive* mutants, as well as *Tet* knockdown) on indirect flight muscle development from the larval to adult stages and during aging in *Drosophila melanogaster*.

**Results:**

We found that *Tet* loss leads to a decrease in the number of adult muscle progenitors in the larva, dysregulation of the myogenic expression program in the pupa and disrupted flight muscle organization in the adult. Interestingly, our data reveal that these phenotypes are largely independent of TET enzymatic activity. However, analysis of TET-catalytic inactive flies also highlights the enzyme’s critical role in adult fly mobility and its ability to prevent premature muscle aging. Further experiments demonstrate that TET expression in muscle progenitors and the central nervous system is essential for maintaining adult mobility.

**Conclusions:**

These results highlight the crucial role of TET beyond 5mC DNA oxidation, suggesting that both catalytic-dependent and catalytic-independent functions of TET are essential for muscle development and function in vivo.

**Supplementary Information:**

The online version contains supplementary material available at 10.1186/s13395-025-00399-x.

## Background

The proper development of the adult muscular system requires a coordinated spatiotemporal regulation of gene expression, spanning from muscle stem cell specification to the terminal differentiation of contractile myofiber bundles [1]. In concert with the sequential activation of specific transcription factors that govern the various steps of myogenesis, chromatin remodelers and epigenetic regulators play a pivotal role in establishing the correct transcriptional program [2, 3]. Notably, cytosine methylation, a widespread epigenetic modification in metazoan genomes, has been implicated in myogenesis regulation. Since the discovery in the late 1970 s that treatments with the DNA demethylating agent 5-azacytidine promote the formation of striated muscles from non-myoblast cells in culture [4, 5], numerous studies have shown that the genome methylation pattern varies during muscle differentiation and aging, as well as in response to environmental cues or between different muscle types [2, 3]. Consequently, the development of drugs targeting the epigenetic enzymes involved in 5-methylcytosine metabolism presents promising potential for regulating muscle growth, regeneration and rejuvenation. However, our understanding of the in vivo functions of these enzymes during myogenesis remains limited, and further research is necessary to fully elucidate their mechanisms of action.

In metazoans, 5-methylcytosine (5mC) is the main form of DNA methylation [6]. This covalent modification is catalyzed by DNA methyltransferase enzymes (DNMT), while members of the Ten-Eleven Translocation (TET) family oxidize 5mC to produce 5-hydroxymethylcytosines (5hmC) and more oxidized derivatives [7]. These intermediates play a dual role, either serving as demethylation intermediates or functioning as distinct epigenetic marks with specific regulatory roles. As a result, TET enzymes have emerged as crucial regulators of 5mC DNA levels and genome expression. They belong to an evolutionarily conserved family of iron and 2-oxoglutarate-dependent dioxygenase found in most metazoans [8]. In vertebrates, TET enzymes are encoded by 3 genes (*TET1*, *2* and *3*) and their corresponding proteins all feature a cysteine-rich domain followed by a bipartite double-stranded ß-helix (DSBH) catalytic domain in their C-terminus. Notably, the long isoforms of TET1 and TET3 also contain an N-terminal CXXC zinc finger domain involved in DNA binding. Vertebrate *TET* genes play both redundant and specific roles in various biological processes [9]. In line with the long-standing hypothesis that DNA demethylation is crucial for activating muscle gene expression, recent studies have implicated TET enzymes in muscle development [10–17]. Although conflicting results have been reported regarding the impact of the loss of *Tet2* on mouse skeletal muscle development in vivo, *Tet2* is critical for adult muscle regeneration [13, 14] and ex vivo experiments suggest that TET2 promotes muscle stem cell proliferation and myogenic differentiation [10, 11, 13–15]. Furthermore, levels of 5hmC, as well as *TET1* and *TET2* expression, increase during myoblast differentiation [10, 11, 15] and several lines of evidence suggest that TET2 activates the expression of key myogenic genes by preventing the methylation of their regulatory regions [10, 11, 13, 14]. Notably, while *Tet1* inhibition does not appear to affect myoblast differentiation in mice [10, 13], it has been proposed that Tet1-induced demethylation contributes to the muscle overgrowth phenotype observed in *Myostatin* mutant cattle [12]. Lastly, *Tet3* loss was found to enhance myoblast differentiation in mice, although the underlying mechanisms remain unexplored [13]. These findings highlight the role of TET enzymes in regulating muscle gene expression ex vivo, but the in vivo functions of these epigenetic regulators during muscle development and aging remain poorly understood.

Importantly, while these studies have focused on the relationship between TET enzymes and 5hmC/DNA demethylation, they have not considered alternative modes of action. Yet these proteins can also exert their effects in a catalytic-independent manner or by oxidizing other substrates, such as m^5^C on RNA [9]. The significance of these non-canonical functions is particularly evident in *Drosophila,* whose genome lacks a 5mC DNA methyltransferase but contains a single *Tet* gene whose expression is essential for adult emergence [18, 19]. In this model organism, TET has been reported to regulate transcription by demethylating 6-methyladenine (6mA) in DNA [18, 20] and to promote mRNA translation by converting m^5^C into hm^5^C [19, 21]. However, recent studies showed that 6mA does not appear to be a substrate for metazoan TET enzymes, even in *Drosophila* [22, 23]. Moreover, TET’s catalytic activity seems dispensable for the development of viable and fertile flies [23, 24]. TET has been found to activate transcription in a catalytic-independent manner in the larval nervous system and the ovarian stem cell niche by facilitating the recruitment of other chromatin regulators [25, 26]. Furthermore, while *Tet null* adult escapers exhibit a “held out” wing phenotype [23, 27], which is often associated with flight muscle defects [28], this defect is not observed in flies lacking TET catalytic activity [23]. These observations suggest that TET might regulate adult myogenesis in a catalytic-independent manner in *Drosophila*. However, its precise role in flight muscle formation remains unexplored.

Here, we have leveraged the presence of a single *Tet* gene and the absence of *DNMT* in *Drosophila* to investigate the 5mC DNA-independent functions of TET during muscle development and aging, focusing on adult indirect flight muscles (IFMs), whose formation has been well characterized [29, 30]. The IFMs arise from adult muscle precursors (AMPs) located in the larval wing imaginal discs. These muscle stem cells are specified during embryogenesis and proliferate in the larval stages to generate thousands of myoblasts [31]. At metamorphosis, the wing disc-associated myoblasts undergo a complex stepwise differentiation process to form multinucleated myofibers organized into bundles containing repeated sarcomere units that drive wing movement in the adult [29, 32]. Using GFP knock-in lines and data mining, we found that TET is expressed in wing disc AMPs and adult flight muscles. Our findings further reveal that TET loss strongly affects IFM formation and AMP numbers in the larval wing disc. However, these phenotypes appear to be largely independent of TET enzymatic activity. Interestingly though, TET catalytic dead mutant flies display mobility defects and signs of premature muscle aging. Moreover, tissue-specific knockdown of TET suggests that its expression is essential both in AMPs and the nervous system for adult mobility. Collectively, our data underscore the significance of TET non-canonical (5mC DNA-independent) functions in regulating muscle development and function.

## Methods

### Fly stocks & crosses

The following *D. melanogaste*r strains were used: *w*^*1118*^ (Bloomington), *Tet*^*null*^*, dTet-Mi (Tet-L-GFP)* [19], *Tet*^*DMAD1*^, *Tet*^*DMAD2*^ [18], *Tet-GFP, Tet*^*CD*^*-GFP* (*Tet*^*CD*^*)* [23], *UAS-RNAi Tet* (BL62280), *control RNAi* (BL36304), *UAS-deGradFP* (BL38421) *UAS-RedStinger* (BL8546), *UAS-mCD8-RFP* (BL27399), *Ubi-GFP, FRT2A* (BL1626), *FRT2A* (BL2024) *kirre-GAL4* (BL66682), *R32D05-GAL4* (BL49712), *nSyb-GAL4* (BL51635) *E(spl)m6-BFM-GAL4* [33], *E(spl)m6-BFM-gapGFP* [34], *Him-GFP* [35]. Unless otherwise specified, crosses and sample collection were performed at 25 °C using classic fly medium (75 g/l organic corn flour, 28 g/l dry yeast, 40 g/l sucrose, 8 g/l agar, 10 ml/l Moldex 20%) with a 12 h dark:light cycle. Mitotic clones were induced using the hsFLP-FRT recombination system [36]. Briefly: *Tet*^*null*^*, FRT2A/TM6B,Tb* males were crossed to *hs-FLP; Ubi-GFP FRT2A* females at 25 °C. Clones were induced in the progeny at 68 ± 4 h after egg laying with a single heat shock at 38 °C for 1 h.

### Immunostaining and confocal imaging

For wing discs immunostaining, wandering third instar larvae were dissected in 1X phosphate-buffered saline (PBS), fixed with 4% formaldehyde in PBS for 30 min, permeabilized in 0.3% Triton X-100-PBS (PBST0.3%) for 30 min and blocked in 1% Bovine Serum Albumin (BSA) 0.1% Triton X-100-PBS (BSA/PBST0.1%) for 1 h. Samples were then incubated overnight at 4 °C with primary antibodies in BSA/PBST0.1%. After three washes of 10 min, samples were incubated for 2 h with secondary antibodies and dyes in PBST0.1% and washed in PBST0.1% three times for 10 min. The samples were mounted in Vectashield for confocal microscopy.

For adult flight muscle immunostaining, adults were anesthetized with FlyNap® (Carolina Biological Supply) and the head, abdomen, wings and legs were removed from the thorax using sharp scissors (Fine Science Tools, No. 15000–02). Thoraces were transferred in 4% PFA in a relaxing solution (20 mM phosphate buffer, pH 7.0; 5 mM MgCl2; 5 mM EGTA, 5 mM ATP) + PBST0.3% and fixed for 30 min at room temperature. Then, thoraces were bisected using a sharp microtome blade (PFM C35, No. 207500003) and fixed again for 20 min. After 3 washes of 5–10 min with PBST0.3%, a standard immunohistochemistry protocol was performed with PBST0.3% for all steps to increase antibody penetration. Primary antibodies were usually incubated over-night at 4 °C, and secondary antibodies for 2 h at room temperature. Stained thorax halves were mounted in Vectashield for confocal microscopy.

For pupal flight muscles, specimens were harvested 0 h after pupal formation (APF) and were left to develop for ~ 96 h before dissection at the pharate stage [37]. The fly was removed from the pupal case before proceeding to thorax isolation and immunostaining as described above for adults.

The following antibodies were used: goat anti-GFP (Abcam ab6673, 1/500), rabbit anti-GFP (Abcam ab6556, 1/500), mouse anti-Fasciclin III (DSHB 7G10, 1/25), rat anti-Kettin (Abcam ab50585, 1/200), mouse anti-Cut (DSHB 2B10, 1/100), rabbit anti-Zfh1 (a kind gift from R. Lehmann, 1/500), rabbit anti-Mef2 (a kind gift from E. Furlong, 1/500), rabbit anti-Twi (a kind gift from K. Jagla, 1/600), mouse anti-Mhc (DSHB 3E8-3D3, 1/50), mouse anti-ATP synthase subunit α (Abcam ab14748, 1/200), mouse anti-α-actinin (DSHB 2G3-3D7, 1/20), rat anti-Elav (DSHB 7E8A10, 1/100) rabbit anti-cleaved Dcp-1 (Cell Signaling Technology #9578, 1/500), mouse anti-Histone H3 phosphoSerine 10 (Abcam #14,955, 1/1000), anti-polyUbiquitin (Sigma-Aldrich FK2, 1/200), donkey anti-rabbit, anti-mouse or anti-goat Alexa Fluor 488 (all from Invitrogen, 1/300), donkey anti-rat or anti-mouse Cy3/Cy5 (Jackson ImmunoResearch, 1/300). Images were acquired using a Zeiss SP8 confocal microscope. For mitochondria observations, images were acquired using a Leica LSM800 confocal microscope with Airyscan. Cell counts were performed with the IMARIS V9.5.0 software. Sarcomere length analyses were performed using MyofibrilJ [38] in Fiji. Neuronal coverage was measured with ImageJ as described for the analysis of tracheal coverage of the midgut [39], but using *nSyb-GAL4*-driven expression of membrane-bound-RFP to label neuronal projections on adult thorax longitudinal sections. Briefly, maximum intensity projections of DLM Z stacks were produced, thresholding was adjusted to ensure the detection of most axonal branches, and the skeleton function was applied. Neuronal coverage was quantified as the number of skeleton pixels divided by DLM surface.

### Micro-computed tomography (µCT) and analysis

The preparation of pupae and adults was performed according to previously published protocols with minor modifications [40]. For adults, flies were anesthetized using CO2 and transferred to 0.5% PBST for 5 min before fixation in Bouin’s solution (5% acetic acid, 9% formaldehyde, 0.9% picric acid; Sigma-Aldrich) for 16–24 h. After several washes in μCT Wash Buffer (0.1 M Na2HPO4, 1.8% sucrose), flies were stained in Lugol’s solution (0.1 N solution of I2KI) for 24–48 h.

The same protocol was used for 96 h APF pupa with a preliminary step to kill the animal and stop the development, which consists of boiling the pupa at 100 °C for 20 s in a heat block, before cooling it to room temperature for 5 min. Then, before adding the fixative, the cuticle of the pupa was poked with a microdissection needle near the anterior and posterior ends to facilitate fixation entry.

After the staining procedure, pupae and adults were washed briefly with water and mounted in a P10 pipette tip filled with water, to allow a 360° sample rotation during scanning. The 3D images were acquired on a Phoenix Nanotom (INRAE, Crouel/Clermont-Ferrand) with a 1 µm pixel size resolution. Image analyses and measurements were performed with IMARIS software, using the surface analysis tool.

### RNA-seq and data analysis

Total RNA was isolated from wing discs of wandering larvae or thoracic flight muscles from 96 h APF pupae dissected in ice-cold PBS, supplemented with RNAlaterTM (Invitrogen) to inactivate RNase, using TrizolTM (Invitrogen) extraction. RNA quality and concentration were assessed with Agilent TapeStation and Qubit devices. Libraries were prepared using Ovation® SoLo RNA-Seq System (Tecan) for *Drosophila* and sequenced by Novogene (Cambridge, UK) on an Illumina Hiseq2000 device (paired-end, 150 bp). Reads were filtered and trimmed to remove adapter-derived or low-quality bases using Cutadapt and checked again with FASTQC. The resulting dataset was deposited on GEO (GSE281163). Illumina reads were aligned to the *Drosophila* reference genome (dm6 Ensembl release 70) with Hisat2. Read counts were generated for each annotated gene using HTSeq-Count. Read normalization, RPKM (Reads Per Kilobase of exon per Megabase of library size), variance estimation and pair-wise differential expression analyses with multiple testing corrections were conducted with an R script using the Bioconductor DESeq2 package. Gene set enrichment analyses were performed with Pangea (PAthway, Network and Geneset Enrichment Analysis) [41], using all the genes expressed (RPKM > 0 in every biological replicate) in the larval wing disc or pupal IFM as a background universe. Enrichment in Gene Ontology terms and Pathways (KEGG, Panther, Reactome) was considered significant for a *p*-value < 0.05 using the Benjamini–Hochberg correction. Predicted protein–protein interaction networks were constructed with STRING (https://string-db.org/) using only experimental and database evidence for connecting nodes and Markov Cluster Algorithm to delineate the main clusters. Publicly available transcriptomic datasets for larval AMPs, pupal and adult IFMs (GSE107247, GSE207241) [38, 42] were analyzed using DESeq2 or RMATS and RATS [43, 44]. Single-cell and single-nuclei RNA-sequencing data for third instar larval wing disc (GSE138626) [45] and adult thorax (GSE227038) [46], respectively, were reanalyzed using the R Seurat v5.2.1 package.

### Western blots

Ten adult thoraces were dissected in ice-cold PBST1% with Protease inhibitor 1X (Roche). Proteins were extracted according to [47]. Briefly, both soluble and insoluble proteins were extracted by first homogenizing the thoraces in PBST1% protease inhibitor 1X before placing them on ice for 10 min. After centrifugation, the supernatant was transferred to a new tube, and a second homogenization was performed on the pellet resuspended in RIPA buffer (5% SDS; 50 mM Tris pH8; 150 mM NaCl; Protease Inhibitor 1X) to extract insoluble proteins. After centrifugation, the second supernatant was pooled with the first one and protein quantification was performed with the DCTM protein assay kit (Biorad) according to the manufacturer’s instructions.

Proteins (50 µg) were diluted in Laemmli sample buffer and denatured at 95 °C for 5 min before being loaded on 4–20% Mini-PROTEAN TGX Stain-Free Gels (BioRad). After migration, proteins were transferred to a nitrocellulose blotting membrane with the BioRad Trans-blot system. Membranes were washed in 1X Tris-Buffered Saline (TBS) and then blocked for 1 h at RT in blocking solution (1X TBS, 10% milk, 0.1% Tween-20). The primary antibodies were diluted in washing buffer (1X TBS, 2% milk, 0.1% Tween-20) and incubated with the membranes overnight at 4 °C at the following concentrations: rabbit anti-Ref(2)P (Abcam ab178440, 1/500), mouse anti-polyUbiquitin (Santa Cruz P4D1, 1/500). Membranes were washed 3 times in washing solution and incubated with the appropriate horseradish peroxidase-conjugated secondary antibodies for 2 h at RT. Detection was performed using the Clarity Western ECL substrate (BioRad) according to the manufacturer’s instructions, and the signals were analyzed with the ChemiDoc MP Imaging System (BioRad).

### ATP quantification assays

Ten adult thoraces were dissected in ice cold PBST1% with Protease inhibitor 1X (Roche). Proteins and ATP were extracted in tissue homogenization buffer (PBS 1X, 100 mM Tris HCl pH8, 4 mM EDTA pH 8, 6 M Guanidine HCl). Samples were incubated at 95 °C for 5 min and centrifuged for 5 min. ATP and protein quantification were performed respectively, with ATP determination kit (Invitrogen) and DCTM protein assay kit (Biorad) according to manufacturer’s instructions. ATP levels were normalized to the amount of proteins.

### mtDNA content analysis

DNA samples were prepared from dissected pupal thoraces at 96 h APF using phenol–chloroform-isoamyl alcohol extraction. QPCRs were performed with SsoFast EvaGreen reagent (BioRad) on a LightCycler 480 Instrument II (Roche Life Science). The following primers were used to amplify mtDNA and gDNA, respectively: *Cyt-b*-F: 5’- CACCTGCCCATATTCAACCAG-3’; *Cyt-b*-R: 5’- TCTTCAACTGGTCGAGCTCC-3’; *Rpl11*-F: 5’-CGAGGGATACCTGTGAGCAGCTT-3’; *Rpl11*-R: 5’-GTCACTTCTTGTGCTGCCATCGT-3’.

### Life span assays

Batches of ~ 10 female adult flies were collected within 8 h of pupal case emergence and transferred every two days to vials containing fresh fly medium. The number of dead flies was recorded every day. A minimum of 100 flies were analyzed per condition.

### Mobility assays

Flight tests were performed as described previously [48]. Around 20 flies were dropped into a 50-cm-long cylinder, covered with a thin layer of oil and divided into 6 zones from top to bottom. The landing height of the flies was then measured. At least 50 flies were scored for each condition. Climbing capacities were assessed using the rapid iterative negative geotaxis (RING) assays [49]. Around 20 flies were transferred to an empty vial and tapped down to induce negative geotaxis. Their progression up the vial was video-recorded and analyzed using Image J. Each vial was tested three times with a 1-min recovery period. The climbing index was measured as the proportion of flies able to climb up to 3 cm in 5 s. At least three vials of flies were scored for each condition. Both climbing and flight tests were performed on flies that had not been anesthetized within the last 24 h.

### Statistical analysis

Violin plots, histograms, volcano plots and GO enrichment dot plots were generated using the R package “ggplot2”. Heatmaps were obtained using the R package Pretty Heatmap (CRAN, pheatmap). Statistical tests were realized with R or Graph Prism 10. Student’s *t*-test was used to compare means between two groups of data, and two-way ANOVA with Tukey’s multiple comparison test was used to compare three or more groups of data. Survival curves were compared using Mantle-Cox log-rank test. Results from flight assays were analyzed using Fisher’s exact test, comparing the repartition of flies between the top and bottom half of the tube in control *versus* test flies. Differences were deemed statistically non-significant with p > 0.05. Statistically significant differences were denoted by * for p < 0.05, ** for p < 0.01, *** for p < 0.001 and **** for p < 0.0001.

## Results

### Tet is expressed in adult flight muscles and their precursors

**Fig. 1:**
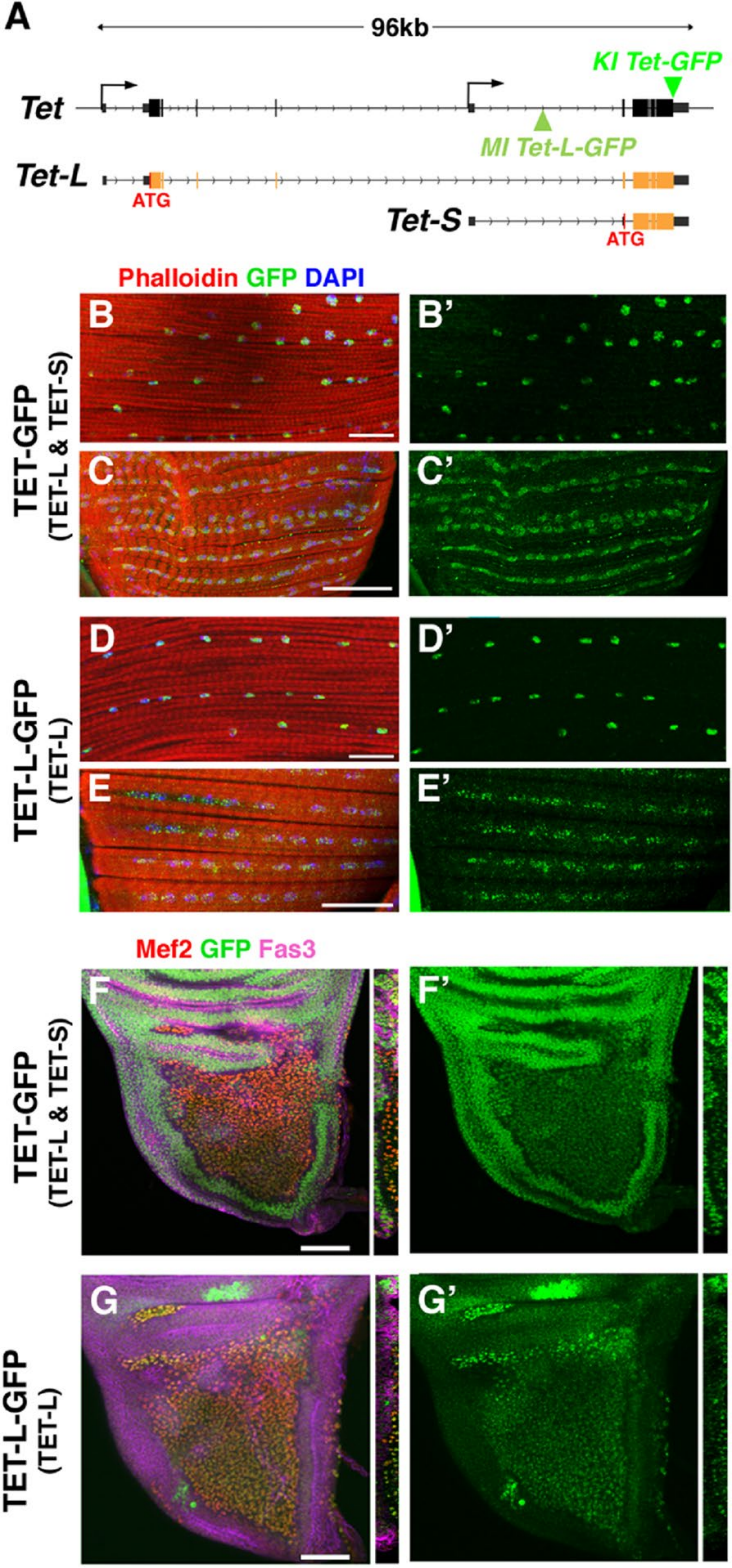
*Tet* is expressed in adult flight muscles and their progenitors. **(A)** Schematic representation of *Tet* gene and its main transcripts. The different exons (black: non-coding; orange: coding) are depicted, as well as the position of the MiMIC insertion (*MI Tet-L-GFP)* [19] and *GFP* knock-in (*KI Tet-GFP)* [23] in the corresponding *Drosophila* lines. The use of two alternate promoters (arrows) leads to the production of distinct mRNAs coding for TET-Long (TET-L) or TET-Short (TET-S). The position of their respective initiation codon (ATG) is shown in red. Of note, the alternative splicing of exon 4 leads to two slightly distinct TET-Long isoforms, and a shorter 3’UTR has been observed for some *Tet-L/Tet-S* transcripts (see Supplemental Fig. 1 for details). The *Tet-L-GFP* line contains a MiMIC insertion with a splice acceptor for an *EGFP-FIAsH-StrepII-TEV-3xFlag* cassette. It allows the incorporation of GFP in TET-Long but not in TET-Short. In *Tet-GFP*, the *GFP* is inserted in frame with the last codon of *Tet*, labelling all isoforms. **(B-E)** Immunostainings against GFP on adult thoraces showing TET-GFP (B, C) or TET-L-GFP (D, E) expression in IFM (B, D) or DFM (C, E). Actin was labelled with phalloidin (red) and nuclei with DAPI (blue). Scale bar: 20 µm. B’-E’: green channel only. **(F, G)** Immunostainings against GFP (green), the AMP marker Mef2 (red) and the epithelial marker Fas3 (magenta) on wing discs of *Tet-GFP* (F) and *Tet-L-GFP* (G) third instar larvae. F’-G’: green channel only. Left panels: top views. Right panels: Z cross sections along the middle part of the notum. Scale bar: 50 µm

To investigate the role of *Tet* in regulating flight muscle development, we first examined its expression pattern at the protein level. *Drosophila Tet* encodes two main protein isoforms generated by alternative promoters (Fig. 1A and Supplemental Fig. 1): TET-Long, which resembles the full-length isoforms of vertebrate TET1/3, and TET-Short, which lacks the N-terminal CXXC domain found in the long isoform, making it similar to vertebrate TET2 or the short isoforms of TET1/3. To assess TET expression in adult flight muscles, we employed two GFP reporters: a *Tet-GFP* knock-in where the GFP is inserted in frame with TET C-terminus, revealing the expression of all isoforms, and a GFP trap *(Tet-L-GFP)* inserted before TET-Short ATG, labelling only TET-Long (Fig. 1A). As shown in Fig. 1B-E, immunostaining for GFP in both reporter lines revealed that TET is expressed in the nuclei of the indirect flight muscles (IFM), which display a characteristic fibrillar organization, as well as in the nuclei of the direct flight muscles (DFM), which exhibit a tubular organization. Furthermore, in the larval wing disc, co-immunolabelling with the AMP marker Myocyte enhancer factor 2 (Mef2) and the epithelial marker Fasciclin 3 (Fas3) demonstrated that TET is expressed in AMP nuclei (Fig. 1F, G). Notably, while TET-L-GFP expression was primarily restricted to AMPs in the notum region and to proneural cells in the wing pouch, as previously reported [27], TET-GFP was detected throughout the wing disc, with higher levels in the underlying epithelial cells. This indicates that the TET-Short isoform has a broader expression pattern than TET-Long in this tissue (Fig. 1F, G, and Supplemental Fig. 1).

To validate these findings and gain further insights into *Tet* expression, we reanalyzed previously published transcriptomic data (Supplemental Fig. 1). A time-course analysis of IFM development revealed that *Tet* expression is highest in third instar larval AMPs and declines as IFM differentiation progresses [38]. Additionally, larval AMPs express similar levels of *Tet-Short* and *Tet-Long* transcripts, while in adult IFM, *Tet-Short* isoforms are more abundant than *Tet-Long* isoforms (Supplemental Fig. 1) [42]. The reexamination of single-cell sequencing data further confirmed that *Tet* is expressed in most AMPs and epithelial cells in third instar larval wing discs [45], as well as in adult thoracic muscles [46]. In summary, *Tet* is expressed throughout IFM development and in mature fibers, with *Tet-Short* isoforms appearing to dominate.

### Tet is required for indirect flight muscle formation

**Fig. 2:**
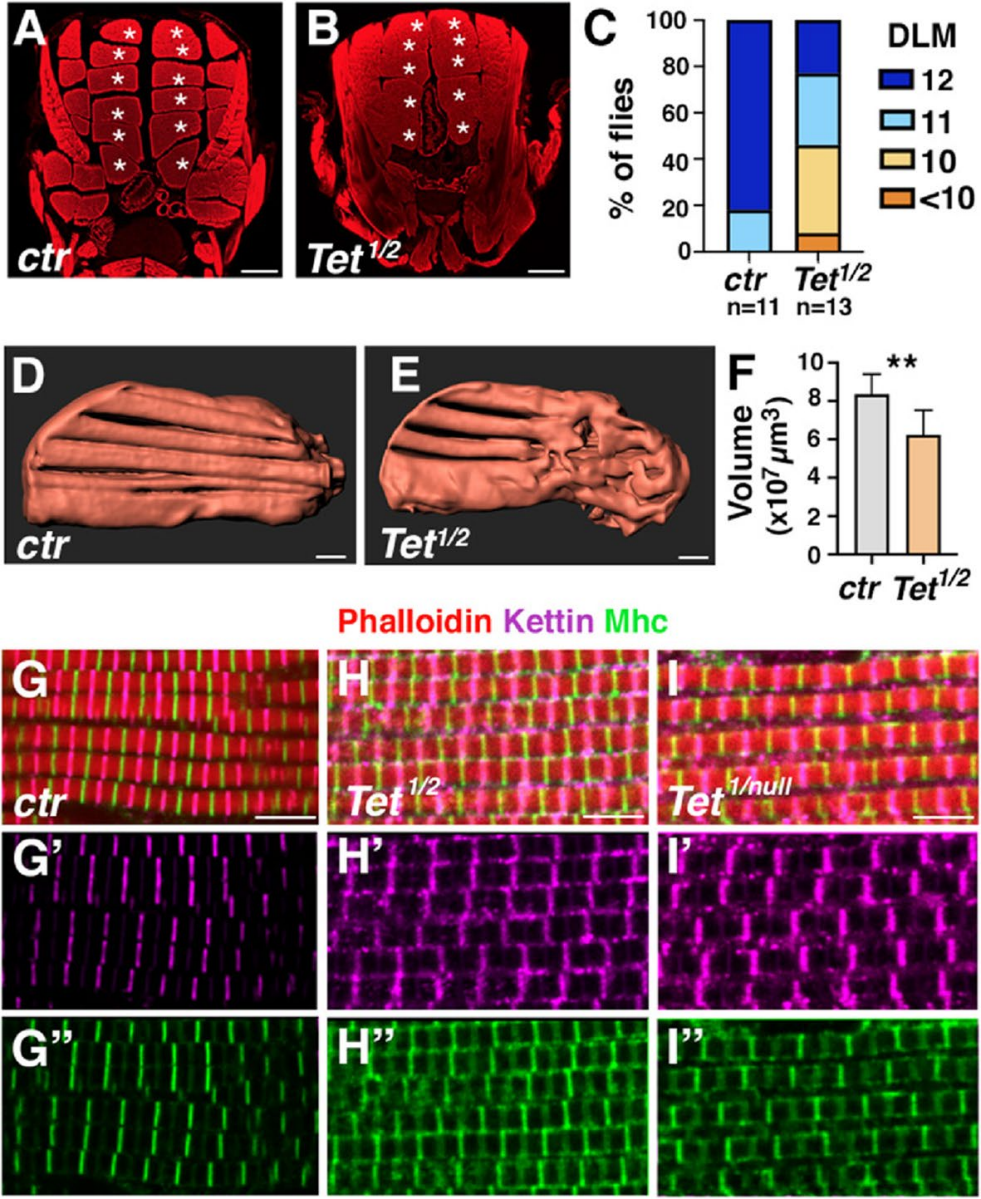
***Tet loss affects IFM formation. (A, B)*** Transverse sections of thoraces of 2-day-old control or *Tet*^*DMAD1/DMAD2*^ (*Tet*^*1/2*^) female flies stained with phalloidin. Individualized DLMs are indicated by a white asterisk. Scale bar: 100 µm. **(C)** Proportion of flies with different numbers of DLM per thorax. The number of flies analyzed for each genotype is indicated at the bottom. **(D, E)** Representative images of DLM 3D reconstruction by microcomputed tomography from whole adult female flies of the indicated genotype. Lateral views; anterior to the left and dorsal to the top. Scale bar: 100 µm. **(F)** Volume of the DLM for each genotype. Data are shown as mean ± SD. ** *P* < 0.01 (Student’s t-test; n = 8 per genotype). **(G-I)** Confocal views of DLM sections stained with anti-Kettin (magenta; G’-I’), anti-Mhc (green; G”-I”) and phalloidin (red) on control (G), *Tet*^*DMAD1/DMAD2*^ (H) *Tet*^*DMAD1/null*^ (I) adult females. Scale bar: 5 µm

Next, we investigated whether *Tet* loss affects IFM development. While flies homozygous for *Tet*^*null*^ (lacking *Tet* mRNA expression) [19], *Tet*^*DMAD1*^ or *Tet*^*DMAD2*^ (which carry premature stop codons before the TET catalytic domain) [18], die at the pupal stage, a small number of *Tet*^*DMAD1/DMAD2*^ individuals give rise to almost immobile adults, display a “held-out” wing phenotype and die within 72 h after hatching [18, 23]. We thus assessed the organization of the IFM in these escapers as compared to age-matched wild-type flies. Phalloidin-stained histological transverse sections revealed six separated bundles of dorsal longitudinal muscles (DLM) in each hemithorax of wild-type flies (Fig. 2A). In contrast, there was a reduction in DLM numbers in *Tet*^*DMAD1/DMAD2*^ adults (Fig. 2B, C), with 50% of the flies showing fewer than 10 DLM per thorax. A slightly more severe phenotype was observed in *Tet*^*null*^ late pupae (96 h APF) (Supplemental Fig. 2). The reduction in DLM number was also apparent on hemithorax sections (Supplemental Fig. 2). In addition, while unruptured myofibers extending across the thorax were present in *Tet*^*DMAD1/DMAD2*^ escapers, some of them exhibited partially dilacerated myofibrils, suggesting that the myofibers are frail. To directly monitor the DLM in situ in adult flies, we took advantage of microcomputed tomography, a non-invasive imaging technique suitable to analyze Drosophila IFM [40, 50]. Thereby, we found that *Tet* mutants exhibited poorly separated myofibrillar bundles and signs of atrophy, along with a significant reduction in muscle volume (Fig. 2D-F, Supplemental Fig. 2, Supplemental Movie 1 & 2). To assess the impact of TET loss on myofibril organization and sarcomere structure, we stained adult thoracic sections with phalloidin and antibodies against Myosin Heavy Chain (Mhc) and Kettin (also known as Sallimus) to visualize the M-bands and the Z-disc, respectively. Confocal imaging revealed a disorganization of the sarcomeres in ~ 20% of *Tet*^*DMAD1/DMAD2*^ adults, as well as in the rare *Tet*^*DMAD1/null*^ escapers we obtained (Fig. 2G-I). Kettin formed discontinuous aggregates at the Z-disc, which were also present in the cytoplasm and at the sarcomere periphery. Mhc localization was similarly affected, with ectopic accumulation at the Z-disc and the sarcomere periphery. Of note, this phenotype was observed on seemingly intact fibers, suggesting that sarcomere protein delocalization is not a consequence of a loss of tension. Furthermore, *Tet* loss was associated with a significant reduction in sarcomere length, while sarcomere thickness was unchanged (Supplemental Fig. 2). These findings indicate that TET is crucial for the proper growth and organization of the IFM.

### Tet loss strongly affects the IFM gene expression program

**Fig. 3:**
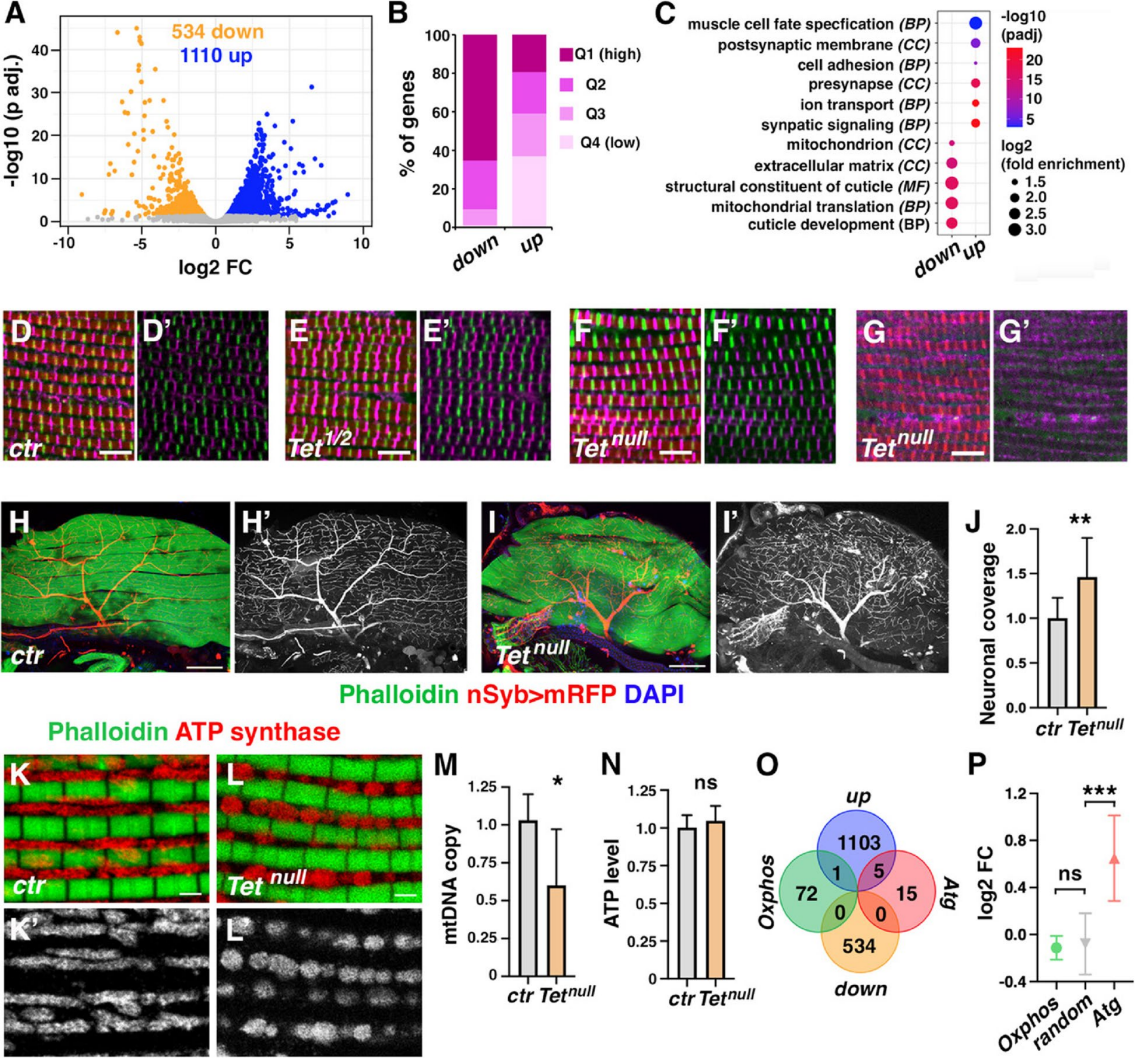
*Tet* is essential for proper gene expression in developing IFM. **(A)** Volcano plot illustrating differentially expressed genes (DEG, adjusted *P* < 0.05, fold change > 1.5) between wild-type and *Tet*^*null*^ conditions as determined by RNA-seq on dissected IFM at the pharate stage. **(B)** Distribution of differentially expressed genes according to their expression quartiles (Q1- > Q4: high—> low expression levels). **(C)** Representative Gene Ontology (GO) categories over-represented among the genes down or upregulated in *Tet*^*null*^ IFM. BP: biological process; CC: cellular component; MF: molecular function. **(D-G)** Confocal views of DLM sections stained with anti-Kettin (magenta), anti-Mhc (green) and phalloidin (red) on control (D), *Tet*^*DMAD1/DMAD2*^ (E), and *Tet*^*null*^ (F, G) 96 h APF pupae. Scale bar: 5 µm. D’-G’: anti-Kettin (magenta), anti-Mhc (green) channels. **(H, I)** Lateral sections of thoraces of control or *Tet*^*null*^ pupae expressing an mRFP protein in neurons (red; *nSyb-GAL4, UAS-mCD8-RFP*), stained with phalloidin (green) and DAPI (blue). Scale bar 100 µm. H’, I’: Red channel only. **(J)** Quantification of neuronal coverage over the DLM**.** ** *P* < 0.01 (Student’s t-test; n ≥ 10 for each genotype). **(K, L)** Confocal views of DLM sections stained with anti-αATP synthase (red) and phalloidin (green) in control (F) or *Tet*^*null*^ (G) pharate pupae. Scale bar: 1 µm. (G’, H’): red channel only. **(M)** Mitochondrial DNA copy number quantification. mtDNA and gDNA contents were measured by qPCR in control or *Tet*^*null*^ IFM. The data are normalized to the control and represent the average ± SD of 6 independent experiments. * *P* < 0.05 (Student’s t-test). **(N)** Quantification of ATP production in control or *Tet*^*null*^ IFM. ns: non-significant (Student’s t-test; n = 4 per genotype).** (O)** Venn diagram showing the overlap between up- or downregulated genes in *Tet*^*null*^ IFM and genes coding for Atg proteins or the Oxphos constituents.** (P)** Differential expression of genes coding for Oxphos constituents, Atg proteins or a random set of genes between *Tet*^*null*^ and wild-type IFM. Means of log2 fold change (log2FC) and 95% confidence intervals are shown. *** *P* < 0.0001 (Mann–Whitney test)

To further investigate the molecular defects associated with *Tet* loss, we established the transcriptome of IFM fibers dissected from 96 h APF wild-type and *Tet*^*null*^ pupae (Supplemental Table 1). We first examined these data by analyzing the top 500 genes with the highest expression levels. Consistent with the previous transcriptomic study of IFM development [38], we observed that in both wild-type and *Tet*^*null*^ samples, these genes were strongly enriched in Gene Ontology terms related to mitochondrial pathways, sarcomere organization or translation (Supplemental Fig. 3; Supplemental Table 2). Also, more than 75% of these genes overlapped with the top 500 expressed genes identified in 90 h APF IFM by Spletter et al*.* [38] (Figure S3). Therefore, our RNA-seq data seem to correctly reflect the transcriptomic state of late-stage developing IFM. Interestingly, differential expression analysis between wild-type and *Tet*^*null*^ samples revealed that *Tet* loss results in the overexpression of 1110 genes and the downregulation of 534 genes (adjusted *P* < 0.05, fold change > 1.5) (Fig. 3A). Hence, *Tet* loss profoundly alters the IFM gene expression program, with a strong bias toward gene activation. Notably, approximately 59% of the upregulated genes are expressed at low levels (Q4 and Q3) in wild-type conditions, compared to only ~ 9% of the downregulated genes (Fig. 3B). In contrast, about 19% of the upregulated genes are expressed at high levels (Q1 quartile), while ~ 66% of the downregulated are highly expressed. These results suggest that *Tet* loss primarily activates genes with low basal expression and dampens those with high expression levels.

Gene ontology and Reactome/KEGG pathway enrichment analyses revealed that upregulated genes were strongly enriched in categories related to synapse organization and signaling and, to a lesser extent, muscle cell fate determination and muscle contraction (Fig. 3C; Supplemental Table 3). This suggests an increased expression of pre- and post-synaptic genes and potentially in the production of neuromuscular junctions, which is known to peak during the late pupal stage [38]. In contrast, downregulated genes were predominantly associated with cuticle formation, extracellular matrix organization, and mitochondrial function (Fig. 3C), the latter being critical for IFM maturation [51]. To further understand the impact of TET loss on IFM, we examined the predicted direct (physical) or indirect (functional) interactions among proteins encoded by the differentially expressed genes using the STRING database [52]. For upregulated proteins, we observed a dense interaction network, with key nodes centered around neurotransmitter reception, synaptic components, and neddylation (Supplemental Fig. 4). Similarly, downregulated proteins formed functional complexes, pointing to a disruption in mitochondrial translation and splicing (Supplemental Fig. 4). In both cases, interactions between components of the network were significantly enriched, with 2.7-fold and 2.3-fold more edges than expected, respectively (*P* < 10^–16^). These findings suggest that *Tet* loss disrupts the expression of functionally related coding genes in the IFM.

**Fig. 4:**
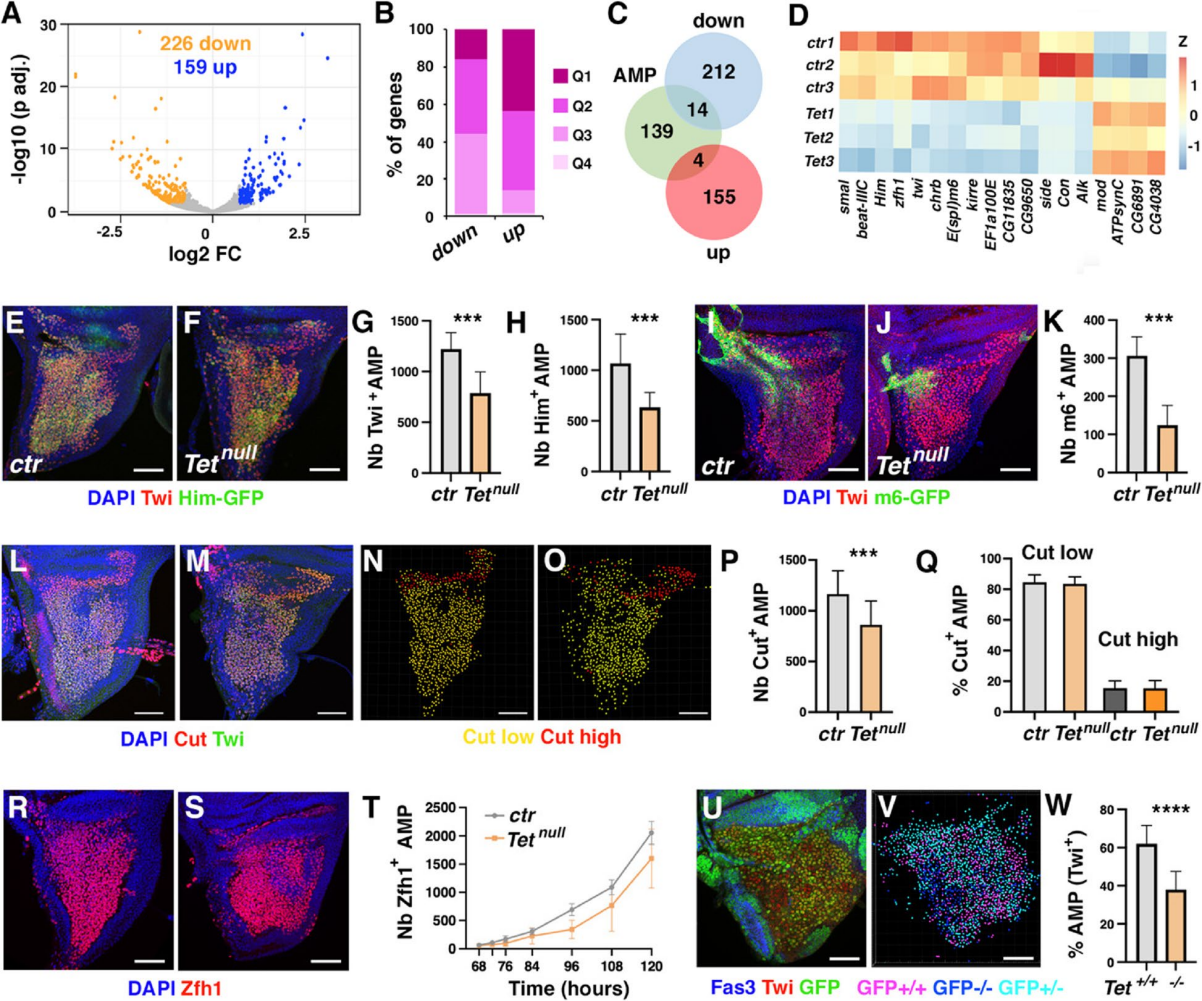
TET regulates AMP numbers. **(A)** Volcano plot illustrating differentially expressed genes (adjusted *P* < 0.05, fold change > 1.5) between wild-type and *Tet*^*null*^ conditions as determined by RNA-seq on third instar larval wing discs. **(B)** Distribution of differentially expressed genes according to their expression quartiles (Q1- > Q4: high—> low expression levels). **(C)** Venn diagram showing the overlap between AMP-specific markers (from single-cell RNA-seq experiment on larval wing discs [45]) and up or downregulated genes in *Tet*^*null*^ wing discs. **(D)** Heatmap showing the expression of the AMP markers affected by TET loss in control (*ctr1, 2, 3*) or *Tet*^*null*^ (*Tet1, 2, 3*) conditions. **(E, F)** Immunostainings revealing the expression of Him-GFP (green) and Twi (red) in the notum region of control (E) or *Tet*^*null*^ (F) third instar larval wing discs. Nuclei were stained with DAPI (blue). Scale bar 50 µm. **(G, H)** Quantification of the number of Twi^+^ (G) or Him-GFP^+^ (H) AMPs. *** *P* < 0.0001 (Student’s t-test; n ≥ 15 for each genotype). **(I, J)** Immunostainings revealing the expression of E(spl)m6-BFM-GFP (green; m6-GFP) and Twi (red) in the notum region of control (I) or *Tet*^*null*^ (J) third instar larval wing discs. Nuclei were stained with DAPI (blue). Scale bar 50 µm. **(K)** Quantification of the number of m6-GFP^+^ AMPs. *** *P* < 0.0001 (Student’s t-test; n ≥ 21 for each genotype). **(L, M)** Immunostainings against Cut (red) and Twi (green) in the notum region of control (L) or *Tet*^*null*^ (M) third instar larval wing discs. Nuclei were stained with DAPI (blue). Scale bar 50 µm. **(N, O)** Corresponding 3-D Imaris-based visualization of the Cut^High^ (red) and Cut^Low^ (yellow) AMP. **(P, Q)** Quantifications of the number of Cut^+^ AMPs (P) and of the proportion of Cut^Low^ *versus* Cut^High^ AMPs (Q). *** *P* < 0.0001 (Student’s t-test; n = 13). **(R, S)** Immunostainings against Zfh1 (red) in the notum region of control (L) or *Tet*^*null*^ (M) third instar larval wing discs. Nuclei were stained with DAPI (blue). Scale bar 50 µm. **(T)** Number of Zfh1^+^ AMPs in control or *Tet*^*null*^ wing discs at different points of larval development. Mean and SD are represented. **(U)** Confocal image showing GFP (green), Twi (red) and Fas3 (blue) expression in the wing disc of *hs-FLP; Tet*^*null*^*,FRT2A/FRT2A,Ubi-GFP* third instar larva carrying clones of *Tet*^*null*^ (GFP^−/−^) or *Tet*^+*/*+^ (GFP^+/+^) cells. Scale Bar 50 µm. **(V)** Corresponding 3-D Imaris-based visualization of the different AMPs (Twi^+^ cells) according to their genotype. **(W)** Quantification of the proportion of wild-type *versus Tet*^*null*^ AMPs. **** *P* < 0.0001 (Student’s t-test; n = 34)

Furthermore, reminiscent of the sarcomere defects observed in the *Tet* mutant, several genes encoding proteins involved in sarcomere assembly or maintenance were deregulated (Supplemental Table 1). Notably, the F-actin regulator Tropomodulin (Tmod) [53], the Z-disc-associated E3 ubiquitin ligase Thin (Tn) [54], and the RNA-binding protein Muscleblind (Mbl) [55] were upregulated. Conversely, Sarcomere length short (Sals), an antagonist of Tmod in thin filament elongation [56, 57], the Z-disc constituents CG1674 [58] and Bicoid stability factor (Bsf) [59], as well as the myosin filament-associated proteins Projectin (encoded by *bent*) [60] and Unc-45 [61] were downregulated. We also observed a downregulation of genes encoding the transcription factors Chorion factor 2 (Cf2) and Zn finger homeodomain 1 (Zfh1), along with Sphingosine-1-phosphate lyase (Sply), all of which are essential for IFM development [50, 62–64]. Accordingly, we assessed whether sarcomere organization was already affected in *Tet* mutant pupae. Immunostainings on hemithorax sections showed that Mhc and Kettin localization was similar to the wild-type in *Tet*^*DMAD1/DMAD2*^ pupae and the majority of the *Tet*^*null*^ mutants (Fig. 3D-F), while ~ 30% of *Tet*^*null*^ pupae presented abnormal fibers with low expression and delocalization of Mhc and Kettin proteins **(**Fig. 3G). This severe phenotype may reflect a defect in sarcomere assembly, or may be caused by muscle degeneration*.*

Since many synaptic genes were upregulated in TET absence, we analyzed DLM innervation using the pan-neuronal driver *nSyb-GAL4* and a *UAS-mCD8-RFP* reporter. As already described [65, 66], in wild-type pharate pupae, the posterior dorsal mesothoracic nerve extended into the DLM from the ventral side and defasciculated into evenly spaced neuronal branches, projecting both anteriorly and posteriorly (Fig. 3H). In contrast, *Tet*^*null*^ thoraces displayed a highly disorganized innervation pattern, with reduced primary branching and convoluted higher-order branches (Fig. 3I), which resulted in a significant increase in neuronal coverage of the DLM (Fig. 3J), indicating that *Tet* is essential for proper DLM innervation.

Furthermore, as our transcriptomic analysis indicates that *Tet* loss impairs mitochondrial ribosome gene expression, we investigated whether it affects mitochondrial production or function. Immunostaining against α-ATP synthase revealed that, in wild-type pupae IFM, mitochondria were densely packed, ellipsoid, and arranged around individual myofibrils. However, in the absence of TET, mitochondria appeared smaller and rounder (Fig. 3K, L). To determine whether this disorganization was linked to a reduced mitochondrial amount, we quantified mitochondrial DNA (mtDNA) content by qPCR. Our results suggest a slight decrease in mtDNA content upon *Tet* loss (Fig. 3M), potentially indicating reduced mitochondrial biogenesis. However, ATP production in dissected thoraces remained unchanged, suggesting that oxidative phosphorylation (OXPHOS) synthesis and activity were not impaired (Fig. 3N). Consistently, the expression of the 73 genes encoding OXPHOS chain components was largely unaffected by *Tet* loss, except for *ND-B8,* which was strongly upregulated (Fig. 3O). The altered mitochondrial morphology may result from dysregulation of the autophagy pathway, a key regulator of mitochondrial network organization [67]. Supporting this hypothesis, 5 out of the 20 genes encoding Atg proteins, including Atg1 or the LC3 homolog Atg8a, were significantly upregulated in TET absence (Fig. 3O). Additionally, the overall expression of the *Atg* gene family increased, whereas OXPHOS gene expression remained unchanged relative to a random gene set (Fig. 3P). These findings suggest that the disruption of the mitochondrial network in IFM may stem from enhanced autophagy gene expression, as shown for *Atg1* overexpression [68].

In summary, our results demonstrate that *Tet* loss induces extensive changes in the expression of interconnected genes critical for IFM maturation.

### Tet regulates AMP development

In light of the above results and considering that IFMs originate from muscle progenitors in the larval wing discs, we conducted RNA-seq analysis on wing discs from wild-type and *Tet*^*null*^ third instar larvae to assess whether AMP development was also impacted. Differential gene expression analyses revealed that *Tet* loss led to the upregulation of 159 genes and the downregulation of 226 genes (adjusted *P* < 0.05, fold change > 1.5) (Fig. 4A, Supplemental Table 4). This suggests that *Tet* plays a relatively mild role in early muscle development compared to its impact at the late pupal stage. In addition, unlike the situation in pupal IFM, *Tet* loss in the larval wing disc primarily suppressed weakly expressed genes and activated strongly expressed ones (Fig. 4B). Specifically, ~ 44% of the repressed genes belonged to the lower two quartiles of expression, compared to only 14% of upregulated genes. Conversely, 43% of activated genes were among the highest quartile of expression, while only 15% of downregulated genes fell into this category. Besides, over 82% of the differentially expressed genes in *Tet*^*null*^ larval wing discs were unaffected in pupal IFMs or the larval brain [25] (Supplemental Fig. 5), indicating that *Tet* primarily influences gene expression in a time- and tissue-specific manner.

**Fig. 5:**
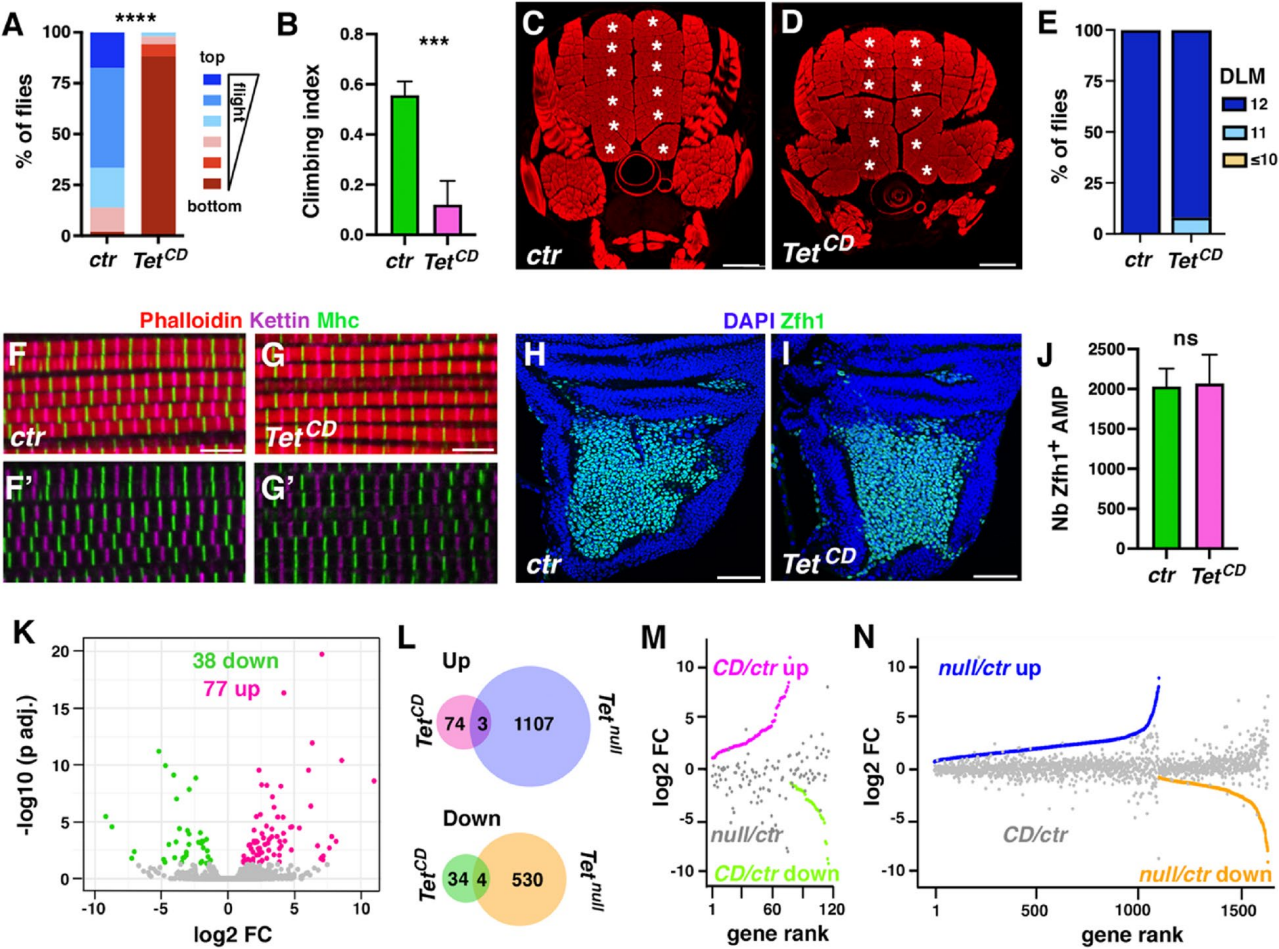
TET enzymatic activity is largely dispensable for flight muscle development. **(A)** Flight capacity of control and *Tet*^*CD*^ adult flies. The percentage of flies landing in each of the 6 zones is indicated. Two batches of at least 50 flies were analyzed for each genotype. *** *P* < 0.001 (Fisher’s exact test). **(B)** Measurement of the climbing capacity of control and *Tet*^*CD*^ adult flies. The climbing index was calculated as a percentage of flies passing a set threshold. *** *P* < 0.001 (Student’s t-test; n = 6 assays, 20 flies per replicate). **(C, D)** Transverse sections of thoraces of 2-day-old control or *Tet*^*CD*^ female flies stained with phalloidin. The individualized DLMs are indicated by a white asterisk. Scale bar: 100 µm. **(E)** Proportion of flies with different numbers of DLM per thorax. Thirteen flies were analyzed for each genotype. **(F, G)** Confocal views of DLM sections stained with anti-Kettin (magenta), anti-Mhc (green) and phalloidin (red) on control (F) or *Tet*^*CD*^ (G) adult females. Scale bar: 5 µm. F’, G’: anti-Kettin (magenta), anti-Mhc (green) channels. **(H, I)** Immunostainings revealing the expression of Zfh1 (green) in the notum region of control (H) or *Tet*^*CD*^ (I) third instar larval wing discs. Nuclei were stained with DAPI (blue). Scale bar 50 µm. **(J)** Quantification of the number of Zfh1^+^ AMPs (n = 20 per genotype). **(K)** Volcano plot illustrating differentially expressed genes (DEG, adjusted *P* < 0.05, fold change > 1.5) between control and *Tet*^*CD*^ conditions as determined by RNA-seq on dissected IFM at the pharate stage. **(L)** Venn diagrams showing the overlap between up (upper panel) or downregulated (lower panel) genes in *Tet*^*CD*^ or *Tet*^*null*^ pupal IFM. **(M)** Representation of the fold changes (log2FC relative to control) of all genes significantly deregulated in *Tet*^*CD*^ (pink: upregulated, green: downregulated) and their respective differential expression in *Tet*^*null*^ (grey). **(N)** Representation of the fold changes (log2FC relative to control) of all genes significantly deregulated in *Tet*^*null*^ (blue: upregulated, orange: downregulated) and their respective differential expression in *Tet*^*CD*^ (grey)

Gene ontology analysis revealed only marginally significant enrichments among deregulated genes. Downregulated genes were primarily associated with extracellular region proteins or carbohydrate binding, whereas upregulated genes were linked to synaptic and cell–cell signaling functions (Supplemental Fig. 5, Supplemental Table 5). Additionally, a STRING-based interaction analysis did not identify hubs among the deregulated proteins (Supplemental Fig. 5). Consequently, these analyses provided little insight into the impact of *Tet* loss on wing disc cells, particularly regarding AMP development.

Since AMPs constitute only a small fraction of wing disc cells, we leveraged a previously published single-cell RNA-seq dataset from larval wing discs [45] to compile a list of 157 AMP markers, *i.e.* genes preferentially expressed in IFM or DFM clusters compared to other cell types (Supplemental Table 6). Notably, 14 of these genes were downregulated and only 4 were upregulated in the absence of *Tet*, indicating a strong enrichment for repressed AMP markers (down: *P* = 4.10^–8^; up: *P* = 0.03; hypergeometric test) (Fig. 4C, D). The downregulated genes comprised several direct targets of the Notch signaling pathway, whose activation is essential for AMP amplification [30]. They included *Enhancer of split m6* (*E(spl)m6-BFM*), *kin of irre* (*kirre)*, *CG9650/Chronophage* (*Cph*), *Holes-in-muscles* (*Him*), *twist (twi)*, and *zfh1* [69], which all regulate AMP differentiation [30, 70–72]. These findings strongly suggest that TET is important for AMP development.

Consistent with this hypothesis, immunostaining for Twi and Him-GFP revealed a significant reduction (35% and 41%, respectively) in the number of AMPs expressing these markers in *Tet* mutant wing discs (Fig. 4E-H). Similarly, we observed a threefold decrease in the number of cells expressing the *E(spl)m6-BFM-GFP* reporter (Fig. 4I-K), which marks a subset of AMPs with high Notch signaling activity [73]. We also assessed the expression of the AMP marker Cut, which is expressed at higher levels in DFM progenitors than in IFM progenitors [74]. We found a decrease in the total number of Cut-expressing AMPs (Fig. 4L-P), but both Cut-high and Cut-low populations were affected, and the proportion of these two populations remained unchanged (Fig. 4Q). Thus, *Tet* seems to affect both populations of AMPs similarly. Next, we monitored the expression of the early AMP marker Zfh1 from 68 to 120 h after egg laying. While the numbers of Zfh1^+^ AMPs were comparable between wild-type and *Tet*^*null*^ larvae in late second instar larvae, their expansion was impaired in the absence of *Tet*, leading to a ~ 25% reduction in Zfh1^+^ AMPs by the wandering third instar larvae (Fig. 4R-T). Since the reduction in AMP number could be due to a decrease in proliferation or an increase in apoptosis, we performed immunostainings against the mitotic marker phospho-histone H3 (pH3) and the apoptotic marker Dcp-1. However, the proportion of mitotic AMPs was not reduced in the absence of TET, and we did not observe any increase in apoptosis in third instar larval wing discs (Supplemental Fig. 5). Finally, to directly assess the cell-autonomous function of TET in AMP/development, we used the FLP/FRT system to generate *Tet*^*−/−*^ clones in early larvae and compared the proportion of wild-type and mutant AMPs (Twi^+^) in third instar wing discs. These experiments showed that *Tet*^*null/null*^ (GFP^−/−^) AMPs were significantly less abundant than their *Tet*^+*/*+^ (GFP^+/+^) counterparts, confirming that *Tet* is required cell-autonomously to regulate AMP numbers (Fig. 4U-W).

### Tet regulates flight muscle development independently of its enzymatic activity

The results above indicate that TET expression is essential for adult flight muscle development. While TET enzymes are best known for modifying 5mC DNA, they can also target m^5^C on RNA or act independently of their enzymatic activity. To determine the functional importance of TET’s catalytic activity, we used a catalytically inactive or “catalytic-dead” mutant allele of *Tet* (*Tet*^*CD*^), introduced in the context of the *Tet-GFP* knock-in flies. Unlike flies lacking TET expression, *Tet*^*CD*^ mutants are viable and fertile [23]. However, *Tet*^*CD*^ flies seemed to have decreased mobility. To confirm this, we performed flight and climbing assays on 1-week old flies. As shown in Fig. 5, *Tet*^*CD*^ adults exhibited severe flight and climbing defects compared to *Tet-GFP* controls (Fig. 5A, B), suggesting that TET enzymatic activity is essential for fly mobility. Despite this, histological analysis of transverse sections in *Tet*^*CD*^ flies revealed no major phenotype in adult flight muscle organization (Fig. 5C, D). Notably, unlike *Tet*^*DMAD1/DMAD2*^ adult escapers (see Fig. 2B, C), *Tet*^*CD*^ mutants did not exhibit a reduction in the number of dorsal longitudinal muscles (DLMs) (Fig. 5E). Furthermore, immunostaining for Phalloidin, Mhc and Kettin showed no localization defect of these proteins within the DLM myofibers (Fig. 5F-H), with sarcomere length and thickness comparable to wild-type (Supplemental Fig. 6). In addition, immunostaining against α-ATP synthase and analysis of nSyb-driven mRFP expression revealed, respectively, that the morphology of the mitochondria and the innervation of the IFM were unaffected in *Tet*^*CD*^ pupae (Supplemental Fig. 6). Also, the loss of TET catalytic activity did not impact AMP numbers in the larval wing disc as measured by immunostaining against Zfh1 or Mef2 (Fig. 5H-J and Supplemental Fig. 6). The fact that the muscle phenotypes observed in the absence of TET expression were not phenocopied in the *Tet*^*CD*^ mutant suggests that TET regulates flight muscle development independently of its catalytic activity.

**Fig. 6:**
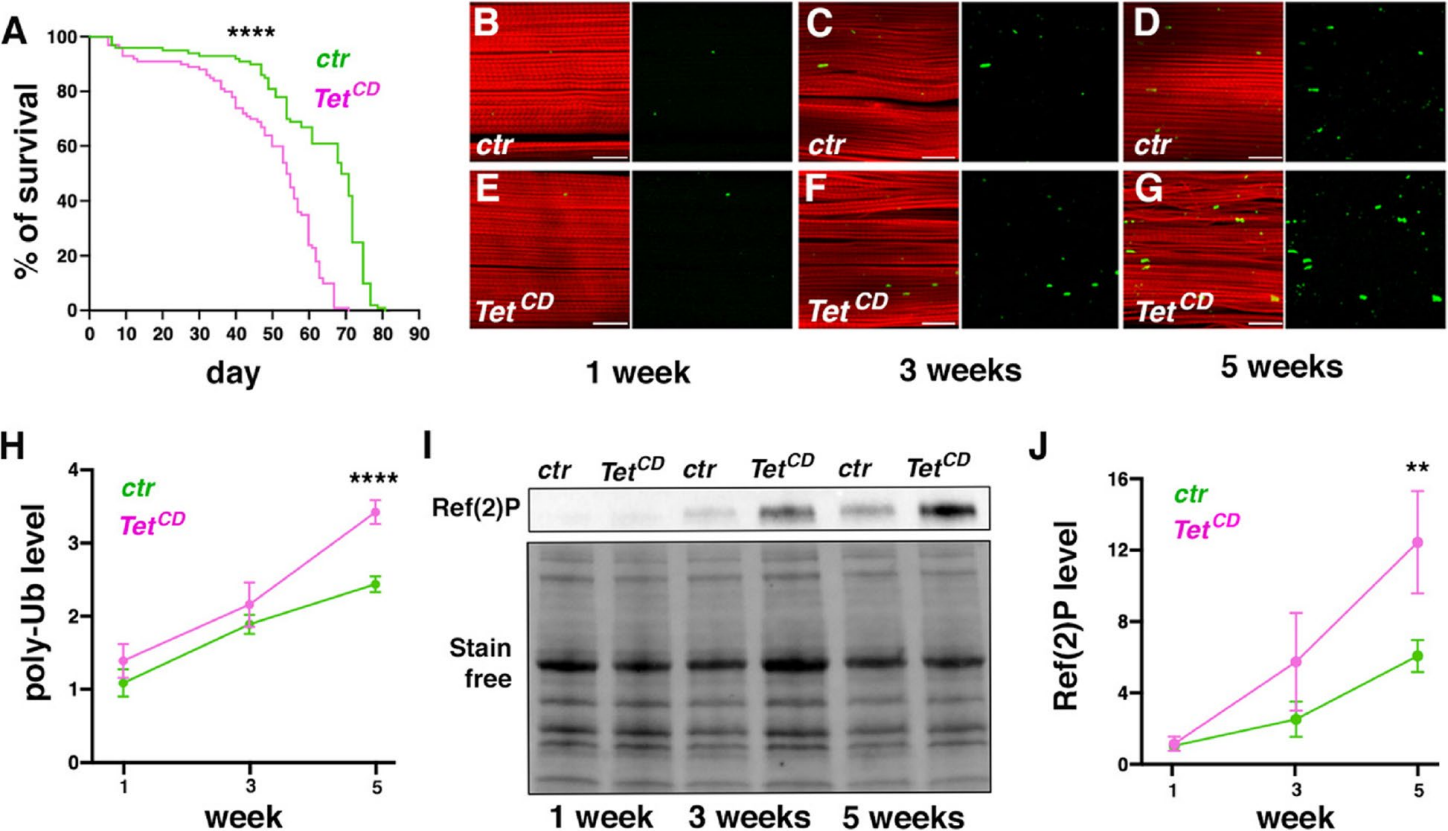
TET enzymatic activity is involved in muscle aging. **(A)** Kaplan–Meier curves showing the lifespan of control and *Tet*^*CD*^ adult flies. **** *P* < 0.0001 (Mantel-Cox; n = 100 per genotype). **(B-G)** Immunostainings against polyubiquitin (green) on adult IFM from control (B-D) or *Tet*^*CD*^ flies (E–G) of the indicated age. Actin was labelled with phalloidin (red). Scale bar: 20 µm. Right panels: green channel only. **(H)** Quantification of polyubiquitin levels in dissected IFM of control (green) of *Tet*^*CD*^ (red) flies of the indicated age as measured by western blot (normalized to total protein amounts). The mean and SD of 4 biological replicates are shown. **** *P* < 0.001 (2-way Anova). **(I)** Western blot showing the expression of Ref(2)P (upper panel) and total protein amounts (stain-free analysis; lower panel) in dissected IFM of control and *Tet*^*CD*^ flies of the indicated age. **(J)** Corresponding quantification of Ref(2)P levels (normalized to total protein amounts). The mean and SD of 4 biological replicates are shown. ** *P* < 0.01 (2-way Anova)

To further assess whether the loss of TET enzymatic activity might affect IFM formation, we established the expression profile of dissected IFM fibers from 96 h APF *Tet*^*CD*^ and *Tet-GFP* control pupae using RNA-seq. As shown in Fig. 5K (and Supplemental Table 7), the absence of TET enzymatic activity had a very limited impact on the IFM gene expression profile: it was associated with the deregulation of only 115 genes (77 upregulated and 38 downregulated genes; adjusted *P* < 0.05, fold change > 1.5). These deregulated genes were not significantly enriched for any GO term or Reactome/KEGG pathway (adjusted *P* < 0.05). Moreover, there was little overlap between the genes either up- or downregulated significantly in *Tet*^*null*^* versus Tet*^*CD*^ IFM (Fig. 5L), and direct comparisons between their fold changes revealed no correlation between these categories of genes (Fig. 5M, N). Hence, it appears that TET primarily regulates gene expression in a catalytic-independent manner in this tissue. Taken together, these findings strongly suggest that while TET expression is essential for IFM development, its catalytic activity is largely dispensable.

### TET catalytic activity plays a key role in preventing flight muscle aging

Since flies lacking TET catalytic activity are homozygous viable, we further analyzed their lifespan and IFM maintenance during aging. Interestingly, *Tet*^*CD*^ flies exhibited a significantly shorter lifespan than their *Tet-GFP* counterparts, suggesting a potential role for TET in delaying aging (Fig. 6A). In *Drosophila*, muscle aging is characterized by the progressive accumulation of protein aggregates [75]. To assess the impact of TET catalytic activity on this process, we monitored the presence of polyubiquitinated protein aggregates in the IFM of 1-, 3- and 5-week-old flies by anti-polyubiquitin immunostaining. Compared to age-matched controls, *Tet*^*CD*^ flies showed a higher accumulation of polyubiquitin aggregates (Fig. 6B-G). This was corroborated by western blots of dissected IFM, which confirmed the age-dependent increase in polyubiquitinated proteins with a significantly stronger accumulation in 5-week-old *Tet*^*CD*^ flies compared to controls (Fig. 6H and Supplemental Fig. 7). It was shown that the accumulation of protein aggregates in aging muscles is associated with an increased level of the autophagy receptor p62/Ref(2)P, which targets ubiquitinated proteins for degradation [76]. In line with this, western blots analysis of dissected IFM revealed an age-dependent increase in Ref(2)P significantly higher in 5-week-old *Tet*^*CD*^ flies than in controls (Fig. 6I, J). Thus, these findings indicate that TET catalytic activity is essential for preventing IFM aging and may contribute to overall fly longevity.

**Fig. 7:**
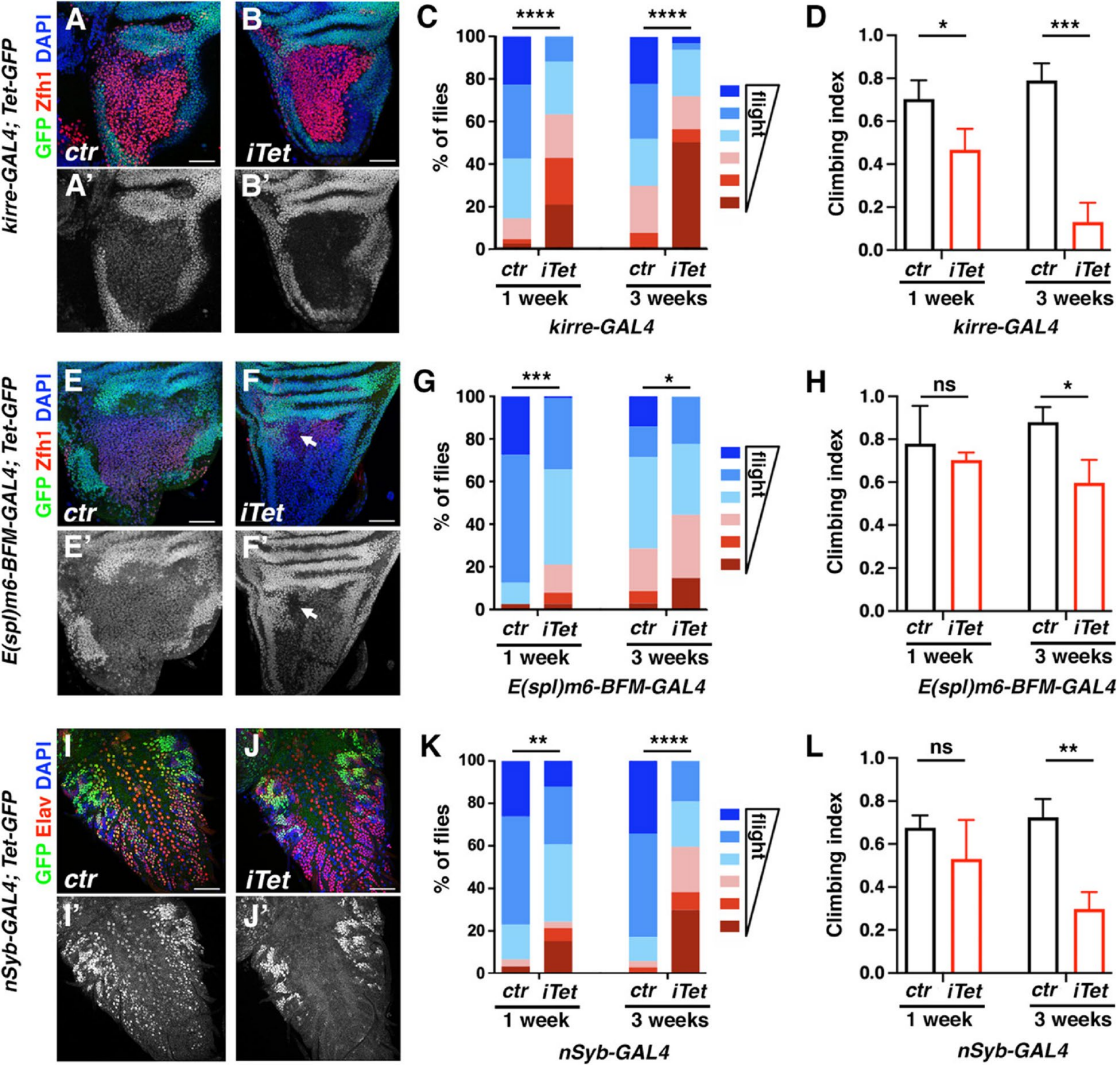
*Tet* expression in the AMPs and the central nervous system contributes to adult fly locomotion. **(A, B)** Immunostaining against GFP (green) and Zfh1 (red) in the wing disc of *Tet-GFP* larvae expressing a control RNAi (A) or an RNAi against *Tet* (B) under the control of the *kirre-GAL4* driver. **(C, D)** Flight test (C) and climbing assay (D) on 1 and 3-week-old flies expressing a control RNAi or an RNAi against *Tet* under the control of *kirre-GAL4. (E, F)* Immunostaining against GFP (green) and Zfh1 (red) in the wing disc of *Tet-GFP* larvae expressing a control RNAi (E) or an RNAi against *Tet* (F) under the control of the *E(spl)m6-BFM-GAL4* driver. **(G, H)** Flight test (G) and climbing assay (H) on 1 and 3-week-old flies expressing a control RNAi or an RNAi against *Tet* under the control of *E(spl)m6-BFM-GAL4. (I, J)* Immunostaining against GFP (green) and Elav (red) in the ventral nerve cord of *Tet-GFP* larvae expressing a control RNAi (I) or an RNAi against *Tet* (J) under the control of the *nSyb-GAL4* driver. **(K, L)** Flight test (K) and climbing assay (L) on 1 and 3-week-old flies expressing a control RNAi or a RNAi against *Tet* under the control of *nSyb-GAL4.* (A, B, E, F, I, J) Nuclei were stained with DAPI. Scale bar 50 µm. Lower panels: GFP (TET-GFP) panel only. (C, G, K) * *P* < 0.05, ** *P* < 0.01, *** *P* < 0.001 (Fisher’s exact test). (D, H, L) The climbing index was calculated as percentage of flies passing a set threshold. The mean and SD of 3 biological replicates are represented. * *P* < 0.05, ** *P* < 0.01, ** *P* < 0.01, *** *P* < 0.001 (Student’s t-test)

### TET expression in the AMPs and the CNS contribute to fly mobility

To further investigate the role of TET in adult fly mobility, we performed RNAi-mediated tissue-specific knockdowns of *Tet*. The induction of *Tet RNAi* in the AMPs using the *kirre-GAL4* driver induced a strong knockdown of TET-GFP expression in these cells in third instar larval wing discs (Fig. 7A, B). The resulting adult flies had strongly impaired flight capacities at 1 week and 3 weeks of age, with more than 50% of flightless flies in the latter case (Fig. 7C). To confirm this result, we took advantage of the DeGradFP method [77] to inhibit TET expression using *Tet-GFP* homozygote flies. Consistent with the RNAi results, *kirre-GAL4*-driven expression of the DeGradFP resulted in an efficient knockdown of TET-GFP expression in AMPs and the resulting adult flies showed a reduced flight capacity (Supplemental Fig. 8). Of note too, the expression of *Tet* RNAi under the control of the AMP-specific driver *R32D05-GAL4* [78] also caused flight deficiency (Supplemental Fig. 8), suggesting that TET expression in the AMPs is important for flight muscle function. In addition, in negative geotaxis assays, 1-week-old *kirre* > *RNAi Tet* flies had reduced climbing performances as compared to controls, and this defect was accentuated in 3-week-old flies (Fig. 7D). With the *E(spl)m6-BFM-GAL4* driver, which is initially expressed in all AMPs but maintained only in a subpopulation of AMPs located in the anterior region of the presumptive lateral hemi-notum [45], we observed a reduction of TET-GFP expression in this location in third instar larvae (Fig. 7E, F). This knockdown was associated with a mild reduction in flight capacity but not in climbing performance in 1-week-old adult flies (Fig. 7G, H). Yet, both flight and climbing defects were observed by 3 weeks (Fig. 7G, H) and were more pronounced in 5-week-old flies (Supplemental Fig. 8). As *Tet* is also strongly expressed in the central nervous system (CNS) and implicated in neuronal differentiation [20, 21, 24, 25, 27, 79], which is crucial for motor function, we also tested the impact of *Tet* knockdown in neurons. Expression of *Tet RNAi* using the pan-neural driver *nSyb-GAL4* effectively reduced TET-GFP expression in the larval CNS neurons as judged by co-immunostaining against GFP and the neuronal marker Elav (Fig. 7I, J). One-week-old *nSyb* > *RNAi Tet* adults had mild flight defects and no significant reduction of climbing index, but 3-week-old flies were strongly affected in both assays (Fig. 7K, L). Altogether, these results suggest that TET expression in the muscle progenitors and neurons is essential for fly mobility and could contribute to its age-dependent decline during *Drosophila* adulthood.

## Discussion

Previous studies have highlighted the role of TET enzymes in myogenesis and muscle regeneration in vertebrates primarily through their regulation of 5mC DNA [10–17]. However, these enzymes also exhibit 5mC DNA-independent functions, whose impact on muscle development remains unexplored. Here, we demonstrate that despite the absence of 5mC deposition machinery in *Drosophila*, TET plays an essential role in both the development and maintenance of adult muscles in this insect. Notably, our findings indicate that while TET regulates IFM development largely in a catalytic-independent manner, its enzymatic activity is essential for fly locomotion and the prevention of IFM aging.

Our analysis reveals that both long and short isoforms of TET are expressed in flight muscle progenitors within the larval wing disc, with *Tet* expression decreasing during muscle differentiation and *Tet-short* being the predominant isoform in the adult. Interestingly, the persistence of TET-Long proteins in adult IFM could be attributed to differences in RNA translation and protein stability, as RNA expression levels do not always correlate with protein abundance [80]. Alternatively, this could result from deregulated *Tet* expression due to the insertion of the Minos element in the *Tet-L-GFP* line [19], which specifically allows the monitoring of TET-Long expression. Developing new tools to investigate TET isoforms might help resolve this issue and provide further insight into the potential functional differences between TET-Short and TET-Long. In this regard, it is worth noting that mammalian *Tet1* and *Tet3* also encode short isoforms lacking the N-terminal CXXC domain [81]. Investigating which TET isoforms are expressed in mammalian muscles and whether they exert distinct functions in this tissue, as observed in other contexts, would be an intriguing avenue for future research [82, 83].

Histological analyses, microcomputed tomography, and immunostainings showed that *Tet* plays a crucial role in adult flight muscle formation, as evidenced by the presence of atrophied IFM with defective sarcomere organization and an altered mitochondrial network. These phenotypes may stem from myogenic developmental defects, as we found that *Tet* is cell-autonomously required in larval AMPs for their amplification, and its knockdown in AMPs impairs adult mobility. The regulation of AMP number by TET was independent of its enzymatic activity and may be linked to defects in Notch signaling, as we observed reduced expression of Notch direct target genes, such as *E(spl)m6-BFM* [69, 84], in *Tet* mutant wing discs. Although AMP proliferation was unaffected in wandering larvae, AMP numbers declined from the late second instar larval stage onward. This suggests that *Tet* influences the early expansion of the AMP pool, which depends on the activation of the Notch receptor by its ligands [31, 73]. Notably, the core components of the Notch pathway were not deregulated at the transcript level in the absence of *Tet*, raising the possibility that TET contributes to Notch-induced gene activation in a catalytic-independent manner. Supporting this hypothesis, recent findings indicate that TET interacts with the chromatin remodeling factor BAP55 to regulate germline stem cell self-renewal independently of its enzymatic activity [26]. BAP55 has also been implicated in the activation of Notch target genes in cell culture [85]. Furthermore, studies in zebrafish suggest that *Tet2/3* are required for Notch-mediated gene activation in the hemogenic endothelium [86]. Although technically challenging, identifying TET target genes and interaction partners in AMPs would provide valuable insights into its molecular mechanism of action.

Besides its impact on AMPs, our transcriptomic profiling of late pupal IFMs indicates that TET expression, but not its enzymatic activity, is essential for the proper expression of numerous genes whose deregulation likely contributes to the muscle defects observed in later stages. However, it is important to note that some *Tet*^*null*^ pupae exhibited severe sarcomere defects and may have already begun muscle degeneration. This could complicate the interpretation of the IFM transcriptome, and it would be interesting to obtain RNA-seq data from earlier time points. Still, we observed a dysregulation of the expression of several genes encoding sarcomere complex components which could contribute to disrupt sarcomere organization [29], while the reduced expression of *Sphingosine-1-phosphate lyase* (*sply*) aligns with the observed decrease in DLM volume [50]. Additionally, the simultaneous upregulation of autophagy-related genes, by disrupting the balance between mitochondrial fission and fusion, could impact the IFM mitochondrial network organization [68], which is itself important for myofibrillogenesis and sarcomere organization [51, 87]. *Tet* loss was also associated with the downregulation of mitochondrial rRNA expression and a slight reduction in mitochondrial DNA content, although ATP levels in the cell remained unchanged. While further experiments on isolated mitochondria would be needed to assess the respiratory chain functions, studies have shown that overexpression of the fission-inducing protein Drp-1 in skeletal muscle can alter the mitochondrial network and reduces mtDNA content without significantly affecting ATP production [88]. Interestingly, *Tet* was also required for the proper expression of components of the Dystrophin/Dystroglycan adhesion complex, including *Dystrobrevin, Sarcoglycan ∂, Syntrophin-like 1* and *Syntrophin-like 2* [89]. Furthermore, TET regulated the expression of *Dyschronic, Lissencephaly-1, SP2353, thin*, *muscle blind* and *sply*, genes previously implicated in *Drosophila* muscular dystrophy models [90–93]. This suggests that *Tet* loss may impair the function of the Dystrophin-associated protein complex (DAPC), which is well known for its involvement in human muscle disorders [89]. Supporting this hypothesis, *Tet* adult escapers frequently displayed an interrupted posterior crossvein (unpublished observation), a phenotype also observed in *dystrophin* and *Dystroglycan* mutants [94]. DAPC dysfunction may further contribute to mitochondrial network disruption and sarcomeric protein mislocalization, as mechanical tension between muscle and tendon cells plays a crucial role in their proper organization [51, 95].

Another salient feature of *Tet* mutant IFM was the upregulation of an interconnected set of genes implicated in synaptic signaling, accompanied by alterations in the innervation network. IFM innervation and neuronal activity are known to influence not only muscle function but also myogenesis [29]. Given that *Tet* knockdown in either neurons or AMPs is sufficient to cause flight and climbing defects, it is plausible that synaptic gene deregulation stems from its role in both cell types, whose development is mutually regulated [65, 66]. Furthermore, it is noteworthy that DAPC disruption within IFMs can also lead to synaptic defects [96]. The concurrent upregulation of both pre- and post-synaptic components suggests an increase in the number of neuromuscular junctions (NMJ), potentially resulting from defects in synapse pruning during early metamorphosis [66]. Alternatively, this could reflect an accumulation of mRNAs encoding synaptic components, which are transported to neuromuscular junctions and whose stability and translation are influenced by synaptic activity [97–99].

A key finding of our study is that TET’s catalytic activity appears largely dispensable for flight muscle development. Notably, the IFM gene expression program was barely affected in the absence of TET’s enzymatic activity, while it was profoundly altered when TET was completely lost. The catalytic-independent function of TET likely depend on its capacity to recruit to chromatin other transcriptional regulators such as BAP55 [26] or PRC1 [25]. Still, *Tet*^*CD*^ flies exhibit severe mobility impairments, and it is tempting to speculate that this phenotype may stem from neuronal defects, as TET catalytic activity contributes to proper gene expression in the CNS [25], and TET-dependent hm^5^C modification on specific mRNAs has been proposed to regulate axon guidance [21]. While the innervation pattern of IFMs did not seem affected in *Tet*^*CD*^ pupae, it would be interesting to test whether the motor neurons function properly. Interestingly, *Tet*^*CD*^ flies also exhibit a shortened lifespan and signs of premature muscle aging. Additionally, mobility defects caused by *Tet* knockdown become more pronounced with age. In the absence of a *bona fide* DNMT in *Drosophila*, TET’s catalytic-dependent functions are likely mediated through the oxidation of m^5^C on RNA. This low abundance epitranscriptomic modification (~ 0.1 to 0.01% of all cytidines) is deposited by RNA methyltransferases such as NSun2 or Mt2, which are conserved across eukaryotes, including in *Drosophila* [100]. While m^5^C is primarily found in tRNAs, it is also detected, albeit at lower levels, in other RNA species, including mRNAs [101]. Notably, m^5^C modifications on tRNAs can influence their maturation, stability, and function, while their oxidation by TET2 has been shown to enhance translation in vitro [102]. Moreover, TET2 depletion affects tRNA fragment accumulation [103], and mounting evidence links tRNA metabolism to aging, including in *Drosophila* [104, 105]. Recent advances in bisulfite-based sequencing should help clarify the impact of TET on m^5^C modifications across different RNA species, even when present at low stoichiometry [106]. Such technological developments are expected to provide deeper insights into TET’s catalytic function and its role in aging.

It is also worth noting that a shorter lifespan and the accumulation of protein aggregates in muscle fibers have been linked to the activation of the Insulin/IGF pathway, which negatively regulates the transcription factor Foxo [107]. Overexpressing Foxo in adult muscle is sufficient not only to prevent muscle degeneration but also to extend fly lifespan [76], and recent snRNA-seq data suggest that Foxo activates *Tet* expression in IFM [46]. Thus, *Tet* may contribute to Foxo protective role in muscle cells. On the other hand, *Tet* expression in insulin-producing cells is essential for axon guidance during brain development [24], and it might also influence aging by altering IGF ligand production by these cells. Given the critical role of inter-organ communication in muscle aging and longevity, the phenotypes observed in *Tet*^*CD*^ flies likely result from a combination of defects in different tissues.

## Conclusions

Our findings show that TET is essential for adult flight muscle development and is essential for maintaining fly mobility. These results underscore TET’s pivotal role in gene regulation, even in the absence of DNA methylation machinery. A similar 5mC-independent mechanism may also underlie TET’s function in vertebrate muscular biology. Although TET’s catalytic activity is not required for muscle development, it is critical for preserving mobility and delaying aging. Identifying the specific substrate(s) of TET in these processes presents an exciting opportunity for future research. Overall, our study highlights the importance of exploring TET’s non-canonical mechanisms of action across species.

## Supplementary Information

Supplementary Material 1: Supplemental Movie 1. µCT-based 3D reconstitution of the DLM in wild-type adult female

Supplementary Material 2: Supplemental Movie 2. µCT-based 3D reconstitution of the DLM in *Tet*^DMAD1/DMAD2^adult female.

Supplementary Material 3: Supplemental Table 1. Differential expression analysis of *Tet*^null^*versus* control dissected IFM in 96 h APF pupae.

Supplementary Material 4: Supplemental Table 2. Gene Ontology enrichments for the 500 genes with highest expression values in IFM dissected 96 h APF (this study) or 90 h APF (Spletter et al. 2018)

Supplementary Material 5: Supplemental Table 3. Gene Ontology term and Reactome or KEGG pathway enrichments for up or downregulated genes in 96 h APF*Tet*^null^ IFM.

Supplementary Material 6: Supplemental Table 4. Differential expression analysis of *Tet*^null^ *versus* control third instar larval wing discs.

Supplementary Material 7: Supplemental Table 5. Gene Ontology term and Reactome or KEGG pathway enrichments for up or downregulated genes in third instar larval *Tet*^null^ wing discs.

Supplementary Material 8: Supplemental Table 6. List of AMP markers derived from Zappia *et al*. (2020).

Supplementary Material 9: Supplemental Table 7. Differential expression analysis of *Tet*^CD^ *versus* control dissected IFM in 96 h APF pupae.

Supplementary Material 10: Supplemental Figure 1. (A) Schematic representation of the *Tet* gene and transcripts. The different exons (black: non-coding; orange: coding) are depicted as well as the position of the MiMIC insertion (*MI Tet-L-GFP)* [19] and *GFP* knock-in (*KI Tet-GFP)* [23] lines. *Tet-RA, -RB, -RE* and *RF* give rise to TET-Long, whereas *Tet-RC* and *-RD* give rise to TET-Short proteins. (B) Schematic representation of the larval wing imaginal disc. The AMPs are located on the surface of the epithelial cells of the notum. (C, D) Immunostainings against GFP on wing discs of *Tet-GFP* (C) and *GFP-Tet* (D) third instar larvae. Scale bar: 50 µm. (E) The expression dynamic of *Tet* during IFM development. Time-course RNA-seq data on developing IFM (GSE107247) [38] were used to analyze *Tet* mRNA levels from the third instar larval stage to adulthood using DESeq2. (F) Relative expression of *Tet* isoforms in third instar larva wing disc-associated myoblasts or 2-day-old adult indirect flight muscles. RNA-seq data from [42] (GSE207241) were reanalyzed using RMATS. (G) Single-cell RNA-sequencing data from [45] (GSE138626) on third instar larval wing disc of wild-type flies were used to analyze *Tet* (left panel), *zfh1* (central panel) and *Fas3* (right panel) expression in individual cells using UMAP dimensional reduction to separate two main population of cells: the AMPs (top left) and the wing disc epithelial cells (bottom right). (H) Single-nuclei RNA-sequencing data from [46] (GSE189214) on adult thoraces of wild-type flies were used to analyze *Tet* (left panel), *Mhc* (central panel) and *sls* (right panel) expression in individual cells using UMAP dimensional reduction to identify muscle cell clusters (*Mhc* and/or *sls*-expressing cells – bottom clusters).

Supplementary Material 11: Supplemental Figure 2. (A, B) Transverse sections of the thoraces of control (A) or *Tet*^null^(B) pharate pupae stained with phalloidin (red). Individualized DLMs are indicated by a white asterisk. Scale bar: 100 µm. (C) Proportion of flies with different numbers of DLM per thorax. The number of flies analyzed for each genotype is indicated at the bottom. (D-E) Hemithorax sections of control (C) or *Tet*^DMAD1/DMAD2^(Tet^1/2^; D, E) adult females stained with phalloidin. The orange arrows indicate the presence of dilacerated myofibrils. Scaler bar: 100 µm. (G, H) Microcomputed tomography images showing a transverse section of the thoracic region of a control (D) or *Tet*^DMAD1/DMAD2^(E) adult female. Scale bar: 100 µm. (I, J) Confocal views of DLM sections stained with anti-a-actinin (red), phalloidin (green) and DAPI in control (H) or *Tet*^DMAD1/DMAD2^(I) adults. Scale bar: 10 µm. (K, L) Measures of sarcomere length (J) and thickness (K) in control or *Tet*^DMAD1/DMAD2^ adult flies. ** *P *< 0.01, ns: not significant (Student’s t-test; n ≥ 12 flies per genotype).

Supplementary Material 12: Supplemental Figure 3. (A) Representative top Gene Ontology (GO) categories over-represented among the 500 most highly expressed genes as detected by RNA-seq on dissected IFM by Spletter *et al.* [38] (90 h APF; GSE107247) and in this study (96 h APF *ctr* or *Tet*^null^). (B) Venn diagrams showing the overlap between the 500 most highly expressed genes as detected by RNA-seq in dissected IFM by Spletter *et al. *[38](blue: 90 h APF; GSE107247) and in this study (red; left panel: 96 h APF *ctr; *right panel: 96 h APF *Tet*^null^).

Supplementary Material 13: Supplemental Figure 4. String-based analysis of the network of physical interactions (sources: text mining, experiments, database) between proteins coded by genes upregulated (A) or downregulated (B) in RNA-seq from dissected IFM from *Tet*^null^ pupae as compared to wild-type flies (96 h APF). The network was refined using MCL-based clustering. Edges between clusters are represented by a dotted line. Only connected nodes are shown. Clusters with at least five components are annotated according to their main function or protein category.

Supplementary Material 14: Supplemental Figure 5. (A, B) Venn diagrams showing the overlap between up or downregulated genes in the absence of *Tet* in third instar larval wing discs (WD), as compared to those deregulated in pupal IFM (A) or in third instar larval central nervous system (CNS) (from Gilbert *et al.* 2024) (B). (C) Main over-represented Gene Ontology terms (BP: biological process; CC: cellular constituent; MF: molecular function) or Reactome pathways (R) among the genes down or upregulated in *Tet*^null^ wing discs. (D, E) String-based analysis of the network of physical interactions between proteins coded by genes upregulated (D) or downregulated (E) in *Tet*^null^ wing discs. Only connected nodes are shown. (F, G) Immunostaining against phospho-Histone H3 (pH3, red) and Zfh1 (green) in control or *Tet*^null^ wing discs. Nuclei were stained with DAPI. Scale bar: 50 µm. (F’, G’): pH3 staining only. (H) Quantification of the proportion of Zfh1^+^ AMP labelled by pH3. (I,J) Immunostaining against Dcp-1 (red) and Cut (green) in control or *Tet*^null^ wing discs. Nuclei were stained with DAPI. Scale bar: 50 µm. (I’, J’): Dcp-1 staining only.

Supplementary Material 15: Supplemental Figure 6. (A, B) Quantification of sarcomere length (A) and thickness (B) in control or *Tet*^CD^adult flies (n ≥ 17 flies per genotype). (C,D) Confocal views of DLM sections stained with anti-aATP synthase (green) and phalloidin (red) in control (C) or *Tet*^CD^ (D) pharate pupae. Scale bar: 1 µm. (C’, H’): green channel only. (E, F) Lateral sections of thoraces of control or *Tet*^CD^ pharate pupae expressing an mRFP protein in neurons (red; *nSyb-GAL4, UAS-mCD8-RFP*), stained with phalloidin (green) and DAPI (blue). Scale bar 100 µm. (E’, F’): Red channel only. (G) Quantification of neuronal coverage over the DLM (n = 10 for each genotype). (H, I) Immunostaining against Mef2 (red) and GFP (green) in control (*Tet-GFP*) and *Tet*^CD^ third instar larval wing discs. Nuclei were stained with DAPI. Scale bar: 50 µm. (J) Quantification of the number of Mef2^+^ AMPs (*n* = 30 per genotype).

Supplementary Material 16: Supplemental Figure 7. (A) Western blot showing polyubiquitin accumulation (upper panel) and total protein amounts (stain-free analysis; lower panel) in dissected IFM of *w*^1118^, *Tet-GFP *and *Tet*^CD^ flies of the indicated age. (B) Corresponding quantifications of polyubiquitin levels (normalized to total protein amounts) as measured in 4 independent experiments. The mean and SD are represented.

Supplementary Material 17: Supplemental Figure 8. (A, B) Immunostaining against GFP (green) and Mef2 (red) in the wing disc of *kirre-GAL4;Tet-GFP/Tet-GFP* (A) or *kirre-GAL4;* *Tet-GFP, UAS-DeGradFP/Tet-GFP* (B) larvae. Nuclei were stained with DAPI. Scale par: 50 µm. (A’, B’): GFP channel only. (C) Flight test on 1-week-old*kirre-GAL4;* *UAS-DeGradFP *(*DeGradFP*)*, kirre-GAL4; Tet-GFP/Tet-GFP* (*Tet-GFP*) or *kirre-GAL4*;*Tet-GFP,UAS-DeGradFP/Tet-GFP* (*Tet-GFP,DeGradFP*) flies.The percentage of flies landing in each of the 6 zones is indicated. At least 50 flies were analyzed for each genotype.** *P *< 0.01 (Fisher’s exact test). (D) Immunostaining against Mef2 (green) in the wing disc of a third instar larva expressing a nuclear RFP (red) under the control of the *R32D05-GAL4* driver. Nuclei were stained with DAPI. Scale bar: 50 µm. (E, F) Flight test on adult flies of the indicated age expressing a control RNAi or an RNAi against *Tet* under the control of *R32D05-GAL4* (E) or* E(spl)m6-BFM-GAL4 *(F).The percentage of flies landing in each of the 6 zones is indicated. At least 50 flies were analyzed for each genotype and time point. **** *P* < 0.0001 (Fisher’s exact test). (G) Climbing assay on 5-week-old flies expressing a control RNAi or an RNAi against *Tet* under the control of *E(spl)m6-BFM-GAL4.* The climbing index was calculated as the percentage of flies passing a set threshold. * *P* < 0.05 (Student’s t-test).

## Data Availability

All relevant data can be found within the article and its supplementary information. RNA-seq data were deposited in GEO (GSE281163).

## Abbreviations

5hmc: 5-Hydroxymethylcytosine
5mC: 5-Methylcytosine
6 mA: 6-Methyladenine
APF: After Pupa Formation
BSA: Bovine Serum Albumin
CNS: Central Nervous System
DAPI: 4’,6-Diamidino-2-phenylindole
DFM: Direct Flight Muscle
DLM: Dorsal Longitudinal Muscle
DSHB: Developmental Studies Hybridoma Bank
hm^5^C: 5-Hydroxymethylcytidine
IFM: Indirect Flight Muscle
m^5^C: 5-Methylcytidine
PBS: Phosphate Buffer Saline
RNAi: RNA interference
TET: Ten-Eleven Translocation
