# Supplementary figures and images for: TET exhibits enzymatic-independent and-dependent functions during Drosophila flight muscle development and aging
Source: Skelet Muscle. 2025 Oct 27;15:30. doi: 10.1186/s13395-025-00399-x (PMC12560471; doi:10.1186/s13395-025-00399-x)

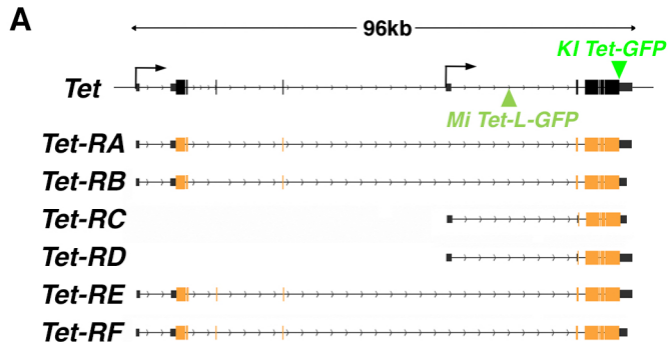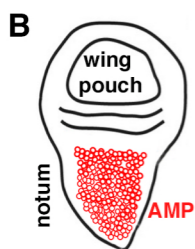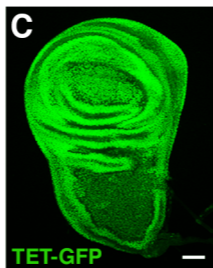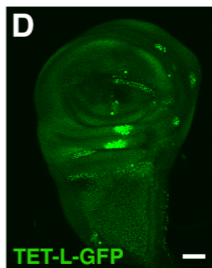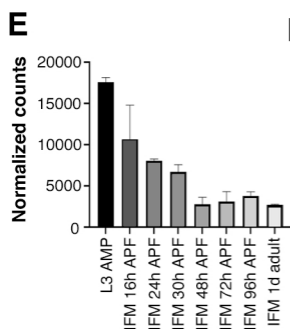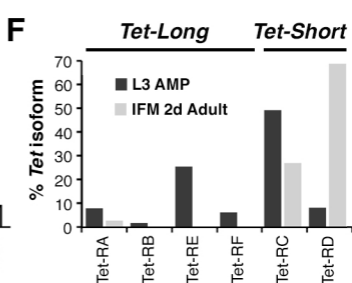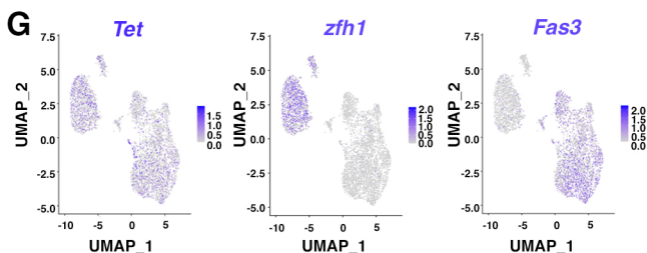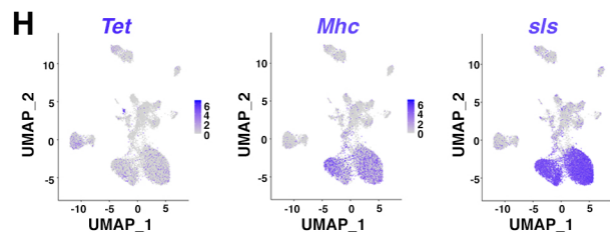

Supplemental Figure 1

Supplement: Supplementary file 10 — Supplementary Material 10: Supplemental Figure 1. (A) Schematic representation of the Tet gene and transcripts. The different exons (black: non-coding; orange: coding) are depicted as well as the position of the MiMIC insertion (MI Tet-L-GFP) [19] and GFP knock-in (KI Tet-GFP) [23] lines. Tet-RA, -RB, -RE and RF give rise to TET-Long, whereas Tet-RC and -RD give rise to TET-Short proteins. (B) Schematic representation of the larval wing imaginal disc. The AMPs are located on the surface of the epithelial cells of the notum. (C, D) Immunostainings against GFP on wing discs of Tet-GFP (C) and GFP-Tet (D) third instar larvae. Scale bar: 50 µm. (E) The expression dynamic of Tet during IFM development. Time-course RNA-seq data on developing IFM (GSE107247) [38] were used to analyze Tet mRNA levels from the third instar larval stage to adulthood using DESeq2. (F) Relative expression of Tet isoforms in third instar larva wing disc-associated myoblasts or 2-day-old adult indirect flight muscles. RNA-seq data from [42] (GSE207241) were reanalyzed using RMATS. (G) Single-cell RNA-sequencing data from [45] (GSE138626) on third instar larval wing disc of wild-type flies were used to analyze Tet (left panel), zfh1 (central panel) and Fas3 (right panel) expression in individual cells using UMAP dimensional reduction to separate two main population of cells: the AMPs (top left) and the wing disc epithelial cells (bottom right). (H) Single-nuclei RNA-sequencing data from [46] (GSE189214) on adult thoraces of wild-type flies were used to analyze Tet (left panel), Mhc (central panel) and sls (right panel) expression in individual cells using UMAP dimensional reduction to identify muscle cell clusters (Mhc and/or sls-expressing cells – bottom clusters). [file 13395_2025_399_MOESM10_ESM.pdf]

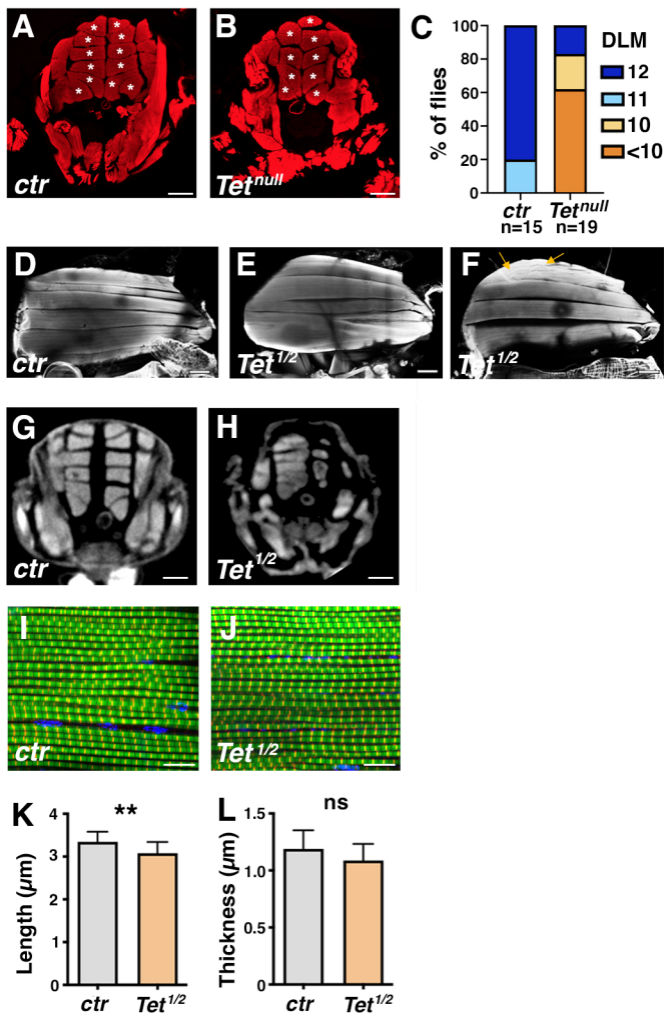

Supplemental Figure 2

Supplement: Supplementary file 11 — Supplementary Material 11: Supplemental Figure 2. (A, B) Transverse sections of the thoraces of control (A) or Tetnull(B) pharate pupae stained with phalloidin (red). Individualized DLMs are indicated by a white asterisk. Scale bar: 100 µm. (C) Proportion of flies with different numbers of DLM per thorax. The number of flies analyzed for each genotype is indicated at the bottom. (D-E) Hemithorax sections of control (C) or TetDMAD1/DMAD2(Tet1/2; D, E) adult females stained with phalloidin. The orange arrows indicate the presence of dilacerated myofibrils. Scaler bar: 100 µm. (G, H) Microcomputed tomography images showing a transverse section of the thoracic region of a control (D) or TetDMAD1/DMAD2(E) adult female. Scale bar: 100 µm. (I, J) Confocal views of DLM sections stained with anti-a-actinin (red), phalloidin (green) and DAPI in control (H) or TetDMAD1/DMAD2(I) adults. Scale bar: 10 µm. (K, L) Measures of sarcomere length (J) and thickness (K) in control or TetDMAD1/DMAD2 adult flies. ** P < 0.01, ns: not significant (Student’s t-test; n ≥ 12 flies per genotype). [file 13395_2025_399_MOESM11_ESM.pdf]

**A**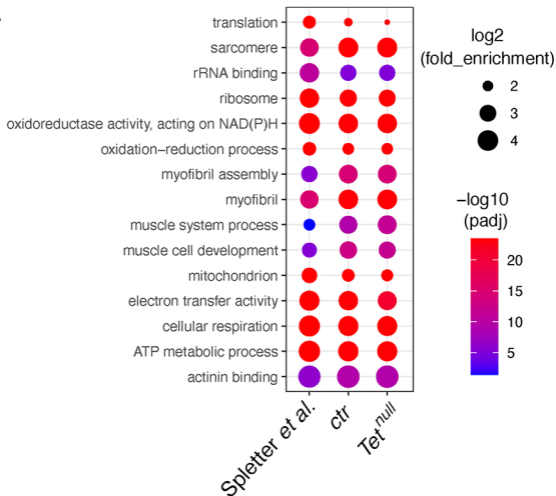**B**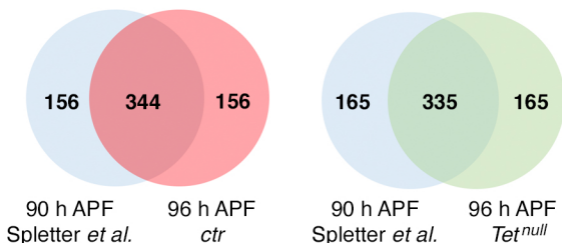**Supplemental Figure 3**

Supplement: Supplementary file 12 — Supplementary Material 12: Supplemental Figure 3. (A) Representative top Gene Ontology (GO) categories over-represented among the 500 most highly expressed genes as detected by RNA-seq on dissected IFM by Spletter et al. [38] (90 h APF; GSE107247) and in this study (96 h APF ctr or Tetnull). (B) Venn diagrams showing the overlap between the 500 most highly expressed genes as detected by RNA-seq in dissected IFM by Spletter et al. [38](blue: 90 h APF; GSE107247) and in this study (red; left panel: 96 h APF ctr; right panel: 96 h APF Tetnull). [file 13395_2025_399_MOESM12_ESM.pdf]

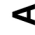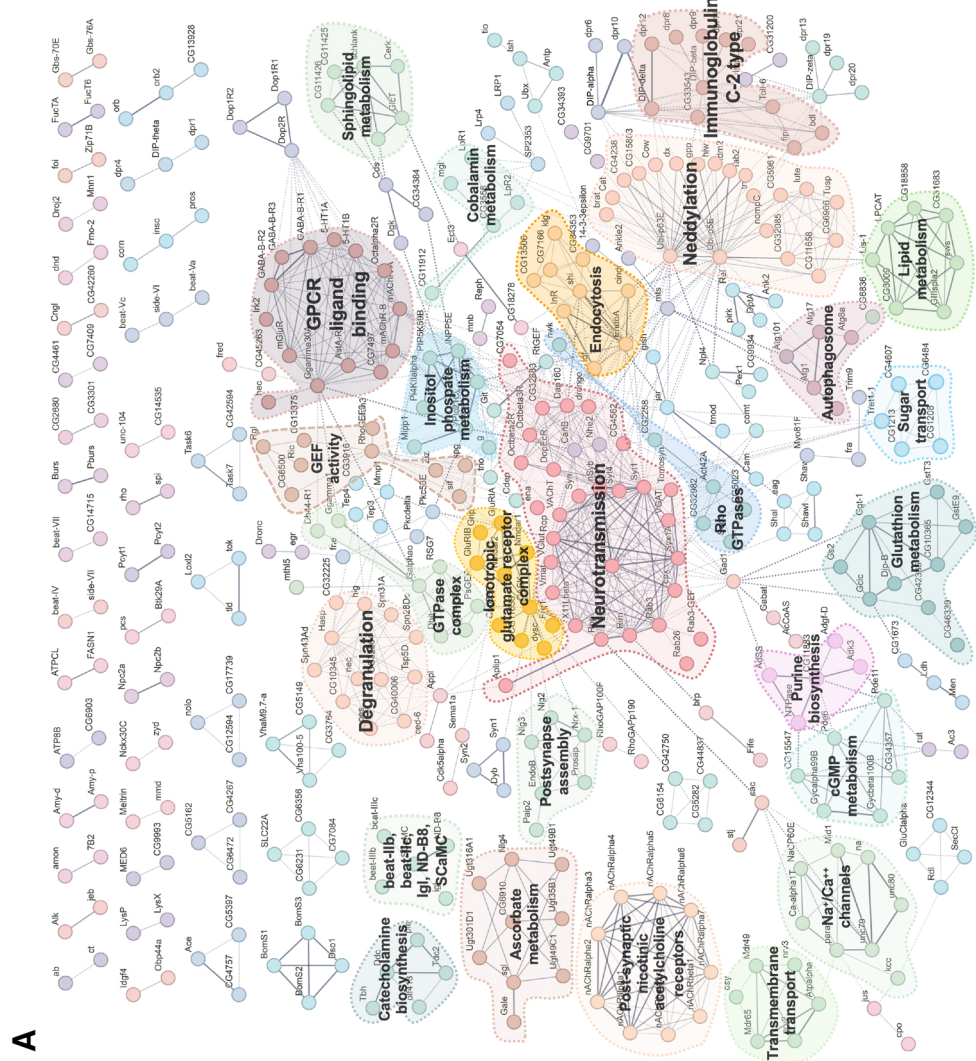

Supplement: Supplementary file 13 — Supplementary Material 13: Supplemental Figure 4. String-based analysis of the network of physical interactions (sources: text mining, experiments, database) between proteins coded by genes upregulated (A) or downregulated (B) in RNA-seq from dissected IFM from Tetnull pupae as compared to wild-type flies (96 h APF). The network was refined using MCL-based clustering. Edges between clusters are represented by a dotted line. Only connected nodes are shown. Clusters with at least five components are annotated according to their main function or protein category. [file 13395_2025_399_MOESM13_ESM.pdf]

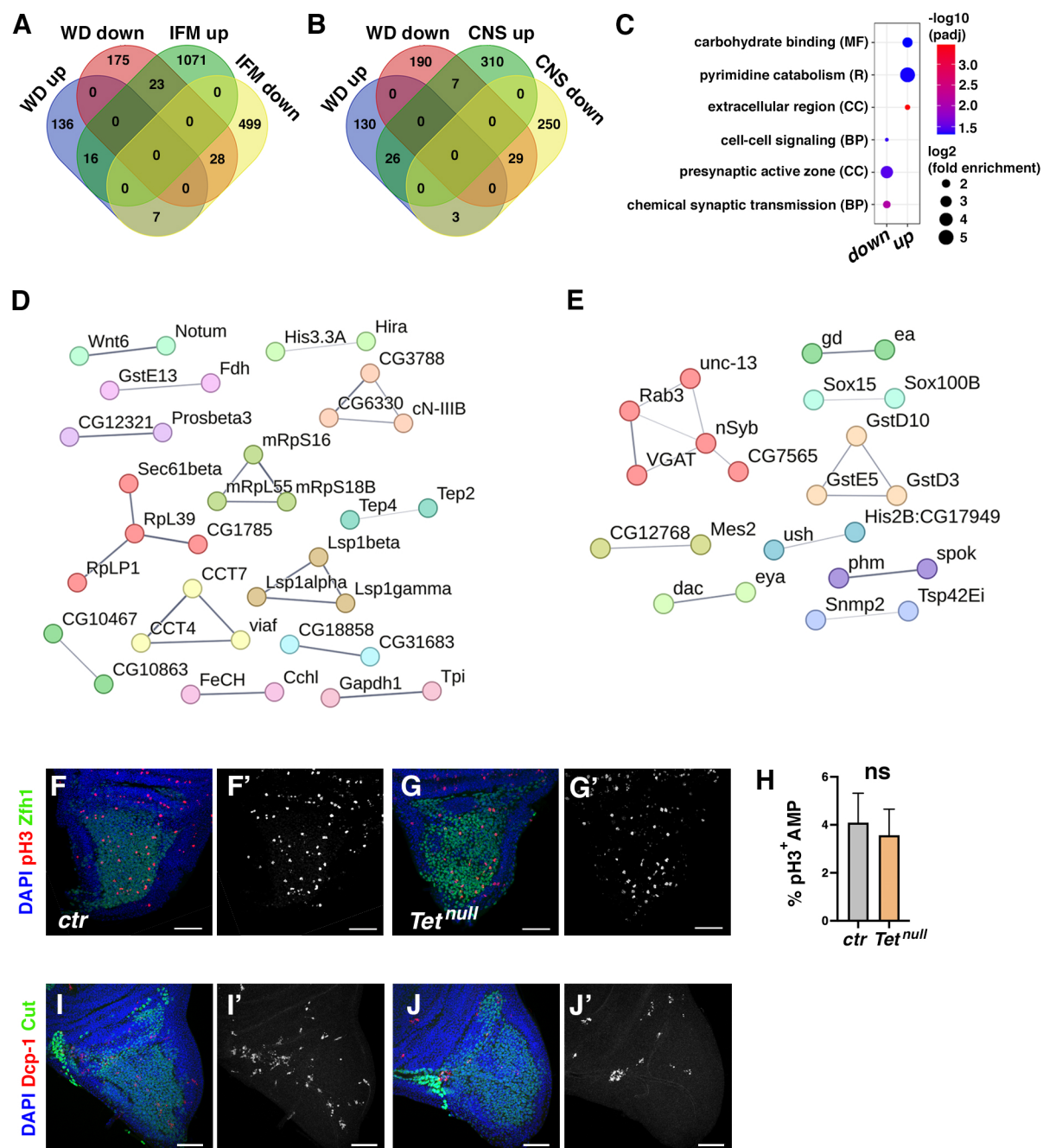

Supplemental Figure 5

Supplement: Supplementary file 14 — Supplementary Material 14: Supplemental Figure 5. (A, B) Venn diagrams showing the overlap between up or downregulated genes in the absence of Tet in third instar larval wing discs (WD), as compared to those deregulated in pupal IFM (A) or in third instar larval central nervous system (CNS) (from Gilbert et al. 2024) (B). (C) Main over-represented Gene Ontology terms (BP: biological process; CC: cellular constituent; MF: molecular function) or Reactome pathways (R) among the genes down or upregulated in Tetnull wing discs. (D, E) String-based analysis of the network of physical interactions between proteins coded by genes upregulated (D) or downregulated (E) in Tetnull wing discs. Only connected nodes are shown. (F, G) Immunostaining against phospho-Histone H3 (pH3, red) and Zfh1 (green) in control or Tetnull wing discs. Nuclei were stained with DAPI. Scale bar: 50 µm. (F’, G’): pH3 staining only. (H) Quantification of the proportion of Zfh1+ AMP labelled by pH3. (I,J) Immunostaining against Dcp-1 (red) and Cut (green) in control or Tetnull wing discs. Nuclei were stained with DAPI. Scale bar: 50 µm. (I’, J’): Dcp-1 staining only. [file 13395_2025_399_MOESM14_ESM.pdf]

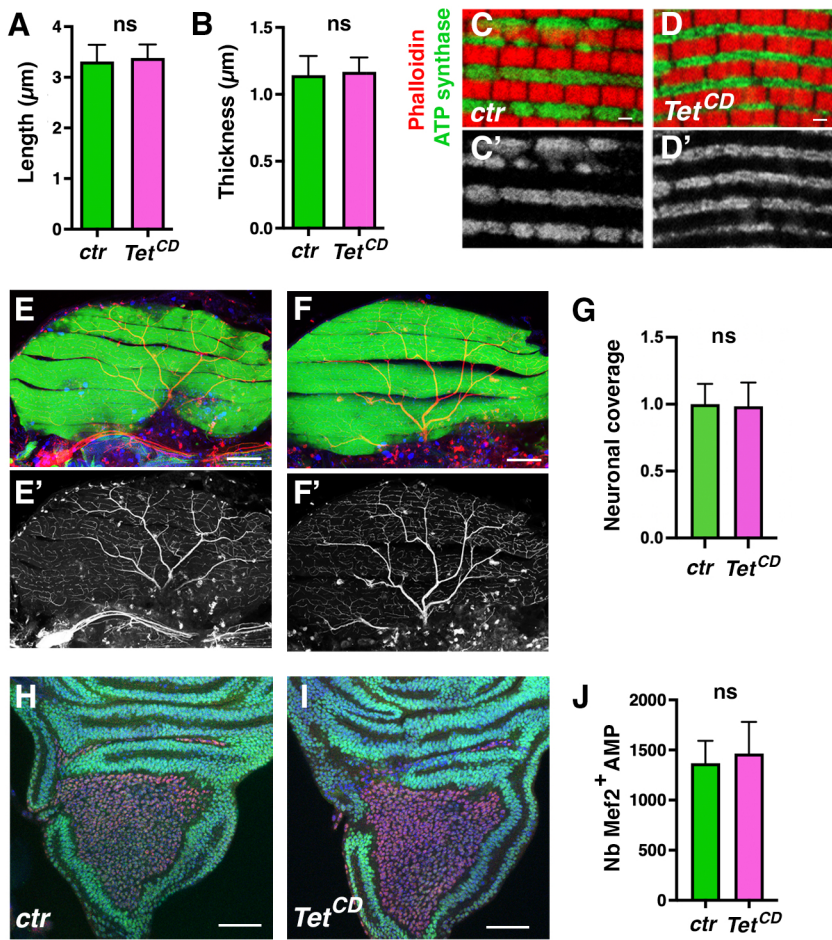

Supplemental Figure 6

Supplement: Supplementary file 15 — Supplementary Material 15: Supplemental Figure 6. (A, B) Quantification of sarcomere length (A) and thickness (B) in control or TetCDadult flies (n ≥ 17 flies per genotype). (C,D) Confocal views of DLM sections stained with anti-aATP synthase (green) and phalloidin (red) in control (C) or TetCD (D) pharate pupae. Scale bar: 1 µm. (C’, H’): green channel only. (E, F) Lateral sections of thoraces of control or TetCD pharate pupae expressing an mRFP protein in neurons (red; nSyb-GAL4, UAS-mCD8-RFP), stained with phalloidin (green) and DAPI (blue). Scale bar 100 µm. (E’, F’): Red channel only. (G) Quantification of neuronal coverage over the DLM (n = 10 for each genotype). (H, I) Immunostaining against Mef2 (red) and GFP (green) in control (Tet-GFP) and TetCD third instar larval wing discs. Nuclei were stained with DAPI. Scale bar: 50 µm. (J) Quantification of the number of Mef2+ AMPs (n = 30 per genotype). [file 13395_2025_399_MOESM15_ESM.pdf]

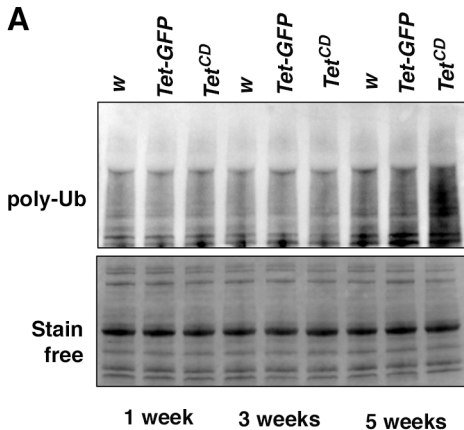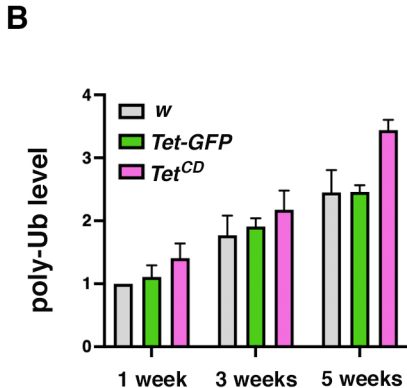

**Supplemental Figure 7**

Supplement: Supplementary file 16 — Supplementary Material 16: Supplemental Figure 7. (A) Western blot showing polyubiquitin accumulation (upper panel) and total protein amounts (stain-free analysis; lower panel) in dissected IFM of w1118, Tet-GFP and TetCD flies of the indicated age. (B) Corresponding quantifications of polyubiquitin levels (normalized to total protein amounts) as measured in 4 independent experiments. The mean and SD are represented. [file 13395_2025_399_MOESM16_ESM.pdf]

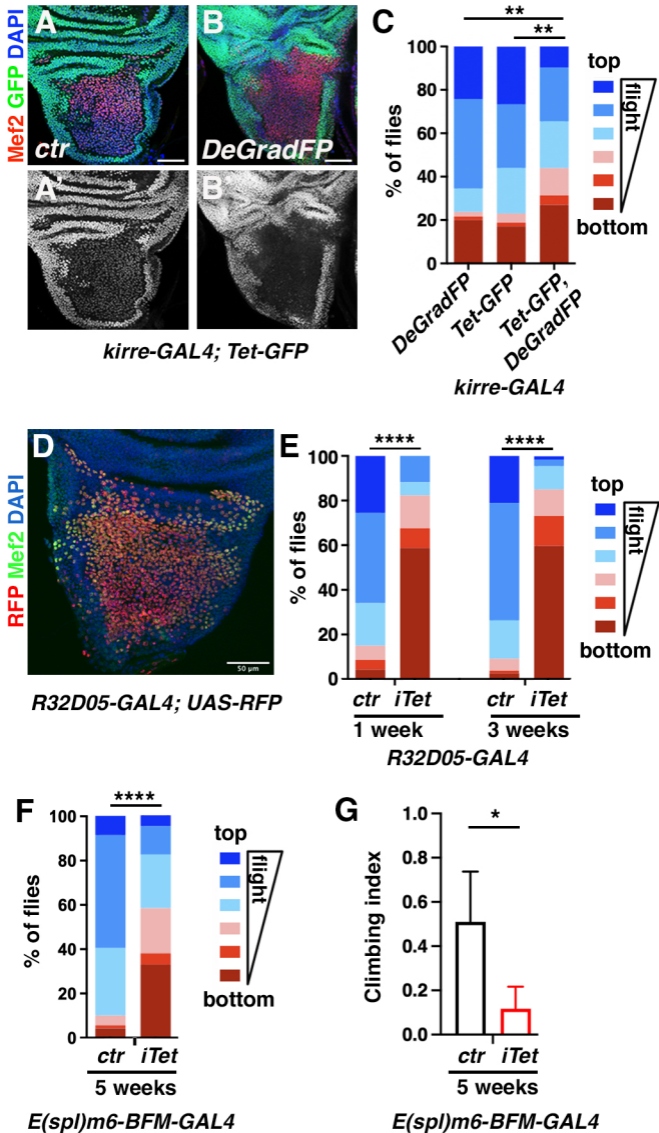

Supplemental Figure 8

Supplement: Supplementary file 17 — Supplementary Material 17: Supplemental Figure 8. (A, B) Immunostaining against GFP (green) and Mef2 (red) in the wing disc of kirre-GAL4;Tet-GFP/Tet-GFP (A) or kirre-GAL4; Tet-GFP, UAS-DeGradFP/Tet-GFP (B) larvae. Nuclei were stained with DAPI. Scale par: 50 µm. (A’, B’): GFP channel only. (C) Flight test on 1-week-oldkirre-GAL4; UAS-DeGradFP (DeGradFP), kirre-GAL4; Tet-GFP/Tet-GFP (Tet-GFP) or kirre-GAL4;Tet-GFP,UAS-DeGradFP/Tet-GFP (Tet-GFP,DeGradFP) flies.The percentage of flies landing in each of the 6 zones is indicated. At least 50 flies were analyzed for each genotype.** P < 0.01 (Fisher’s exact test). (D) Immunostaining against Mef2 (green) in the wing disc of a third instar larva expressing a nuclear RFP (red) under the control of the R32D05-GAL4 driver. Nuclei were stained with DAPI. Scale bar: 50 µm. (E, F) Flight test on adult flies of the indicated age expressing a control RNAi or an RNAi against Tet under the control of R32D05-GAL4 (E) or E(spl)m6-BFM-GAL4 (F).The percentage of flies landing in each of the 6 zones is indicated. At least 50 flies were analyzed for each genotype and time point. **** P < 0.0001 (Fisher’s exact test). (G) Climbing assay on 5-week-old flies expressing a control RNAi or an RNAi against Tet under the control of E(spl)m6-BFM-GAL4. The climbing index was calculated as the percentage of flies passing a set threshold. * P < 0.05 (Student’s t-test). [file 13395_2025_399_MOESM17_ESM.pdf]
